# Atomistic machine learning with irreducible Cartesian natural tensors

**DOI:** 10.1038/s41467-026-77263-z

**Published:** 2026-08-27

**Authors:** Qun Chen, A. S. L. Subrahmanyam Pattamatta, Boyu Wang, David J. Srolovitz, Mingjian Wen

**Affiliations:** 1 Institute of Fundamental and Frontier Sciences, University of Electronic Science and Technology of China, Chengdu, China; 2 Department of Mechanical Engineering, The University of Hong Kong, Hong Kong SAR, China

**Keywords:** Atomistic models, Computational science, Computational methods

## Abstract

Atomistic machine learning is a powerful tool for accurate and efficient investigation of material behavior at the atomic scale. While attempts have been made to construct models directly within Cartesian space, they face challenges in providing a systematic framework based on irreducible representations—a core feature of widely used spherical models. Here we propose Cartesian Natural Tensor Networks to overcome these limitations and thus offer a general, symmetry-preserving framework for atomistic machine learning. We present a theory of irreducible representations using Cartesian natural tensors, comprising their construction, their products, and a systematic scheme to decompose and reconstruct high-rank physical tensors. Leveraging this machinery, we develop equivariant machine learning interatomic potentials for materials and molecular systems with performance on par with leading spherical models. It further captures accurate structure–property relationships for tensorial quantities ranging from low-rank dipole moments to high-rank tensors with complex symmetries, such as the elastic constant tensor.

## Abstract (web summary)

A theory of Cartesian tensors gives rise to CarNet, a unified machine learning framework that predicts the properties of molecules and materials with leading accuracy, from interatomic potential energies to complex, high-rank elastic tensors.

## Introduction

Atomistic machine learning (ML) represents a data-driven paradigm that learns to predict material and molecular properties directly from the atomic structure. The primary inputs are the spatial coordinates of atoms and their chemical identities, while the outputs span interatomic potential energies and forces^1,2^; scalar properties such as bond dissociation energies and band gaps^3,4^; and tensorial quantities including dipole moments, polarizabilities, and elastic constant tensors^5,6^. When trained on quantum-mechanical data, these models approach ab initio accuracy at a fraction of the computational cost, enabling simulations of larger systems and longer time scales, and systematic exploration across vast chemical and materials spaces. By uniting accurate physical data with scalable learning approaches, atomistic ML underpins transferable interatomic potentials, rapid property screening, and the construction of robust structure–property relationships. Atomistic ML has become foundational in the sciences and engineering, accelerating rational discovery and design across diverse fields, including energy conversion and storage^7,8^, heterogeneous catalysis^9,10^, and pharmaceutical discovery^11,12^.

At the core of any atomistic ML model lies the central task of describing the local atomic environment around each atom. This step is critical to any atomistic ML because it converts raw atomic coordinates and chemical identities into numerical representations that ML algorithms can process. Early methods relied on descriptors designed to capture important structural features like bond lengths and bond angles (e.g., atom-centered symmetry functions^13^, the Coulomb matrix^14^, and DeePMD descriptors^15^). More recently, the field has evolved toward more systematic approaches based on mathematical expansions of the atomic environment using well-established basis functions. Current state-of-the-art approaches use two key mathematical tools: polynomials and/or trigonometric functions to describe distance relationships between atoms, and spherical harmonics to capture angular relationships. Examples include SNAP^16^, ACE^17^, TFN^18^, NequIP^19^, MACE^20^, and GRACE^21^. In other words, these methods first transform atomic structures from their Cartesian coordinates (i.e., {x, y, z} positions) into spherical coordinates (distances and angles), and then train the ML model in this spherical representation. However, since atomic structures are naturally described in Cartesian coordinates for most simulation methods and property calculations, there is a compelling motivation to develop methodologies that work directly in Cartesian space, directly utilizing geometric information in its natural form while maintaining clear physical interpretability.

Considerable progress has been made in developing atomistic ML methods that operate directly in Cartesian space. For example, models such as MTP^22^, REANN^23^, CACE^24^, CAMP^25^, and ICTP^26^ build on the ideas of Cartesian atomic moments or Cartesian cluster expansion to construct interatomic potentials. FieldSchNet^27^, TensorNet^28^, HotPP^29^, and TACE^30^ utilize Cartesian representations to predict low-rank tensorial properties. Despite these achievements, Cartesian approaches still face key challenges that limit their broader applicability. First, models employing reducible Cartesian tensors, such as HotPP^29^ and CAMP^25^, naturally accommodate arbitrary-rank tensors and respect global rotational symmetry, but do not systematically enforce the internal index symmetries of physical tensors (e.g., *C*_*ijkl*_ = *C*_*jikl*_ = *C*_*klij*_ of the elastic constant tensor), leaving unphysical redundancies in the predicted tensors. Second, models employing irreducible Cartesian representations, such as TensorNet^28^, ICTP^26^, and TACE^30^, could in principle be extended to higher ranks, but in practice remain limited to low-rank tensorial properties due to the absence of a systematic scheme for the decomposition and reconstruction of physical tensors using irreducible Cartesian building blocks. These limitations motivate a principled and systematic framework to fully harness the advantages of working directly in Cartesian space.

This work addresses these challenges by developing a comprehensive computational framework for atomistic ML using Cartesian natural tensors. A natural tensor is one whose components are determined by the geometric properties of the space in which it lives and is, therefore, an intrinsic geometric construction rather than a regular tensor. Natural tensors find wide application in fluid physics (e.g., Navier–Stokes equations)^31^, electromagnetism (e.g., multipole expansions of charge distributions)^32^, and increasingly in machine learning (e.g., equivariant neural networks)^26,28^. In this work, we adapt the theory of irreducible Cartesian natural tensors for atomistic ML, further develop a systematic scheme for the decomposition and reconstruction of arbitrary physical tensors, and provide the mathematical tools and software needed to apply this framework to atomic systems.

Building on this foundation, we introduce a modeling framework applicable to both interatomic potentials and structure–property relationships of high-rank tensorial observables with intrinsic symmetries. Our Cartesian Natural Tensor Networks (CarNet) framework is E(3)-equivariant; i.e., it respects the three-dimensional rotational, translational, and inversion symmetries inherent to atomic systems. We demonstrate the capabilities of CarNet through a range of atomistic ML tasks. As an MLIP, CarNet achieves accuracy comparable to leading spherical-tensor approaches across both materials and molecular systems, with universal models that generalize across the periodic table. Beyond potentials, CarNet provides a unified route to predicting tensorial properties of varying rank and symmetry, including dipole moments, polarizabilities, nuclear magnetic shielding, and elastic constant tensors. CarNet enables the exploration of the rich landscape of Cartesian representations and their applications in atomistic ML.

## Results

### Cartesian natural tensor theory

A natural tensor is a fully symmetric Cartesian tensor whose traces vanish on any pair of indices^33–35^. Formally, a rank-*n* tensor **X**_*n*_ with components ${X}_{{i}_{1}{i}_{2}\ldots {i}_{n}}$ is natural if it is

1. Symmetric: ${X}_{{i}_{\pi (1)}{i}_{\pi (2)}\ldots {i}_{\pi (n)}}={X}_{{i}_{1}{i}_{2}\ldots {i}_{n}}$ for all *π* ∈ *S*_*n*_, where *S*_*n*_ is the symmetric group of degree *n* (the group of all permutations of *n* indices),
2. Traceless: ${\delta }_{{i}_{a}{i}_{b}}{X}_{{i}_{1}\ldots {i}_{a}\ldots {i}_{b}\ldots {i}_{n}}=0$ for all 1 ≤ *a* < *b* ≤ *n*,

where *δ*_*ij*_ is the Kronecker delta and Einstein summation is implied over repeated indices (the same below unless otherwise stated). Scalars (rank-0 tensors) and vectors (rank-1 tensors) are trivially natural tensors.

**Fig. 1:**
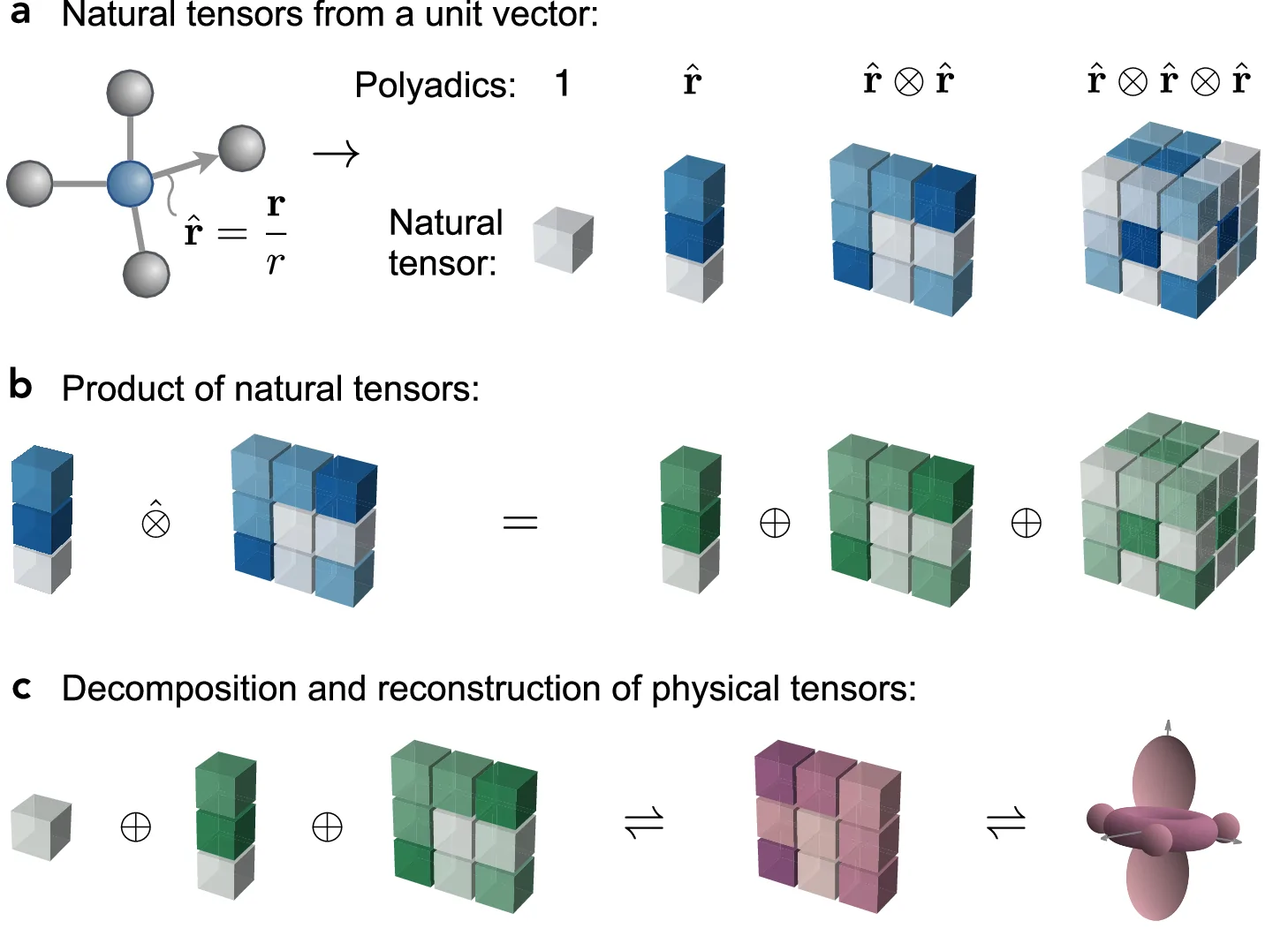
Schematic illustration of Cartesian natural tensor operations. **a** Construction of natural tensors of different ranks from a unit vector $\hat{{{{\bf{r}}}}}$. **b** Tensor product between a rank-1 and a rank-2 natural tensor generates three natural tensors of ranks 1, 2, and 3. **c** A physical tensor (e.g., the nuclear shielding tensor) can be decomposed into a set of natural tensors and, conversely, reconstructed from them. ⊗ denotes the product between ordinary tensors, $\hat{\otimes }$ denotes the product between natural tensors, and ⊕ denotes the direct sum of natural tensors.

Natural tensors are widely used to represent physical properties. For example, any rank-2 Cartesian tensor **T** (*T*_*ij*_) can be decomposed into

1 $${T}_{ij}=\underbrace{\frac{1}{3}{T}_{kk}{\delta }_{ij}}_{n=0}+\underbrace{\frac{1}{2}\left({T}_{ij}-{T}_{ji}\right)}_{n=1}+\underbrace{\frac{1}{2}\left({T}_{ij}+{T}_{ji}\right)-\frac{1}{3}{T}_{kk}{\delta }_{ij}}_{n=2},$$

where the *n* = 0, 1, 2 natural tensors are typically called the isotropic, antisymmetric, and symmetric parts, respectively (Fig. 1c). This decomposition is useful because each part has a distinct physical meaning. For example, if **T** is a stress tensor, the isotropic part represents the hydrostatic pressure (causing volumetric change), the antisymmetric part is related to torques (zero in classical continuum mechanics), and the symmetric part represents the shear stress (causing shape change). Conversely, given three natural tensors of ranks 0, 1, and 2, one can fully reconstruct a rank-2 Cartesian tensor.

In three dimensions, a rank-*n* natural tensor **X**_*n*_ has 3^*n*^ components, but only 2*n* + 1 of these are independent (due to the symmetry and traceless constraints). A rank-*n* natural tensor **X**_*n*_ furnishes a 2*n* + 1-dimensional irreducible representation of the special orthogonal group SO(3) (the group of 3D rotations). Intuitively, under rotations, the 2*n* + 1 independent components mix in a way that prevents decomposition into smaller sets of components that transform independently under SO(3); formally, the representation space of **X**_*n*_ is irreducible under SO(3), meaning it contains no proper nontrivial SO(3)-invariant subspaces^36^. This makes natural tensors particularly well-suited for atomistic ML, where rotational equivariance is crucial. Below, we summarize the three fundamental natural tensor operations underlying our framework: constructing natural tensors from a unit vector, computing products of natural tensors, and decomposing and reconstructing physical tensors. Further details are provided in Supplementary Note 1.

Given a unit vector $\hat{{{{\bf{r}}}}}$, a rank-*n* natural tensor **X**_*n*_ can be constructed in two steps (Fig. 1a). 1. Symmetric polyadic tensor: Form the rank-*n* tensor ${{{\bf{U}}}}={{{{\bf{r}}}}}^{\otimes n}=\hat{{{{\bf{r}}}}}\otimes \hat{{{{\bf{r}}}}}\otimes \cdots \otimes \hat{{{{\bf{r}}}}}$ (repeated *n* times). By construction, **U** is fully symmetric. 2. Trace removal: Project **U** into the symmetric traceless subspace via **X**_*n*_ = **H** ⊙ ^*n*^**U**, where **H** is the rank-2*n* projection tensor, and  ⊙ ^*n*^ denotes *n*-fold contraction over *n* pairs of indices (i.e., ${X}_{{k}_{1}\ldots {k}_{n}}={H}_{{k}_{1}\ldots {k}_{n}{i}_{1}\ldots {i}_{n}}{U}_{{i}_{1}\ldots {i}_{n}}$). **H** is given by^37^

2 $${H}_{{k}_{1}\ldots {k}_{n}{i}_{1}\ldots {i}_{n}}={\sum}_{t=0}^{\lfloor n/2\rfloor }{(-1)}^{t}\cdot F\cdot \{{\delta }_{ki}^{\otimes n-2t}{\delta }_{kk}^{\otimes t}\}{\delta }_{ii}^{\otimes t},$$

where $F=\frac{(2n-2t-1)!!}{(2n-1)!!}$, the superscript ^⊗*t*^ is a shorthand notation for the *t*-fold tensor product of Kronecker deltas (e.g., ${\delta }_{ki}^{\otimes 2}={\delta }_{{k}_{1}{i}_{1}}{\delta }_{{k}_{2}{i}_{2}}$ and ${\delta }_{ii}^{\otimes 2}={\delta }_{{i}_{1}{i}_{2}}{\delta }_{{i}_{3}{i}_{4}}$), and the curly braces { } denote symmetrization achieved by summing over all unique permutations of the indices. For example, when *n* = 2, we have ${H}_{{k}_{1}{k}_{2}{i}_{1}{i}_{2}}={\delta }_{{k}_{1}{i}_{1}}{\delta }_{{k}_{2}{i}_{2}}-\frac{1}{3}{\delta }_{{k}_{1}{k}_{2}}{\delta }_{{i}_{1}{i}_{2}},$ and the corresponding rank-2 natural tensor is ${X}_{{k}_{1}{k}_{2}}={H}_{{k}_{1}{k}_{2}{i}_{1}{i}_{2}}{U}_{{i}_{1}{i}_{2}}={r}_{{k}_{1}}{r}_{{k}_{2}}-\frac{1}{3}{\delta }_{{k}_{1}{k}_{2}}{r}_{{i}_{1}}{r}_{{i}_{1}}.$ The projection tensor **H** is analogous to spherical harmonics that generate irreducible representations of SO(3) from a unit vector, but it is expressed entirely in Cartesian form.

The tensor product between two natural tensors can be expressed as a direct sum of a set of natural tensors^35,38^. Given natural tensors ${{{{\bf{X}}}}}_{{l}_{1}}$ of rank *l*_1_ and ${{{{\bf{Y}}}}}_{{l}_{2}}$ of rank *l*_2_, their product

3 $${{{{\bf{Z}}}}}_{{l}_{3}}={{{{\bf{X}}}}}_{{l}_{1}}\hat{\otimes }{{{{\bf{Y}}}}}_{{l}_{2}}$$

is a tensor whose rank lies in the range ∣*l*_1_ − *l*_2_∣ ≤ *l*_3_ ≤ *l*_1_ + *l*_2_ (analogous to spherical tensors^39^), where $\hat{\otimes }$ represents the natural tensor product (e.g., when *l*_1_ = 1 and *l*_2_ = 2, ${{{{\bf{Z}}}}}_{{l}_{3}}$ is of rank 1, 2, or 3; see Fig. 1b). To obtain ${{{{\bf{Z}}}}}_{{l}_{3}}$, we can first compute the ordinary tensor product ${{{\bf{W}}}}={{{{\bf{X}}}}}_{{l}_{1}}\otimes {{{{\bf{Y}}}}}_{{l}_{2}}$, and then symmetrize **W** and remove the traces. Similar to the construction of natural tensors from unit vectors, based on the formulas in ref. ^38^, we have derived an explicit expression for a rank-(*l*_1_ + *l*_2_ + *l*_3_) projection tensor **H** that performs this operation. For even *l*_1_ + *l*_2_ − *l*_3_ = 2*d*, we have

4 $$	{H}_{{k}_{1}\ldots {k}_{{l}_{3}}{i}_{1}\ldots {i}_{{l}_{1}}{j}_{1}\ldots {j}_{{l}_{2}}}=\hfill\\ 	{\sum}_{t=0}^{\min ({l}_{1},{l}_{2})-d}{(-2)}^{t}\cdot F\cdot \{{\delta }_{ik}^{\otimes {l}_{1}-(d+t)}{\delta }_{jk}^{\otimes {l}_{2}-(d+t)}{\delta }_{kk}^{\otimes t}\}{\delta }_{ij}^{\otimes d+t},$$

where $F=\frac{(2{l}_{3}-2t-1)!!}{(2{l}_{3}-1)!!}$. For odd *l*_1_ + *l*_2_ − *l*_3_ = 2*d* + 1, the formula is similar but with the multiplication of an additional Levi–Civita symbol (expression given in Supplementary Note 1). With this, ${{{{\bf{Z}}}}}_{{l}_{3}}$ can be computed as ${{{{\bf{Z}}}}}_{{l}_{3}}={{{\bf{H}}}}{\odot }^{{l}_{1}}{{{{\bf{X}}}}}_{{l}_{1}}{\odot }^{{l}_{2}}{{{{\bf{Y}}}}}_{{l}_{2}}$ (i.e., ${Z}_{{k}_{1}\ldots {k}_{{l}_{3}}}={H}_{{k}_{1}\ldots {k}_{{l}_{3}}{i}_{1}\ldots {i}_{{l}_{1}}{j}_{1}\ldots {j}_{{l}_{2}}}{X}_{{i}_{1}\ldots {i}_{{l}_{1}}}{Y}_{{j}_{1}\ldots {j}_{{l}_{2}}}$). Here, the projection tensor **H** is analogous to the Clebsch–Gordan coefficients^39^ for spherical tensor product.

A central task in utilizing natural tensors involves converting between regular Cartesian tensors and their natural representations. The decomposition and reconstruction in Eq. (1) for rank-2 tensors are possible for arbitrary tensors; however, the complexity increases significantly with the rank and internal index symmetries of the tensor. Unlike the rank-2 case in Eq. (1), higher-rank tensors yield multiple linearly dependent decomposition candidates^34^, which should be carefully selected to form a linearly independent basis set of natural tensors. Moreover, the internal index symmetries of a physical tensor impose additional constraints on the form of natural tensors^37^. For example, a generic rank-4 tensor yields six rank-3 natural tensor candidates in its decomposition spectrum, but only three of them are linearly independent. The rank-4 elastic constant tensor **C** has symmetries *C*_*ijkl*_ = *C*_*jikl*_ = *C*_*klij*_, under which all associated rank-3 candidates vanish. Several studies have examined the decomposition of physical tensors with specific ranks^37,40–42^; a more general approach was proposed by Coope et al.^33–35^. However, a general method for tensors of arbitrary rank and symmetry remains unavailable (to our knowledge). We have developed a systematic approach to addressing the challenges, which consists of three key steps.

First, create the rank-(*m* + *n*) tensor **G** to map between the rank-*n* physical tensor space and the rank-*m* natural tensor space^34^:

5 $${G}_{{k}_{1}\ldots {k}_{m}{i}_{1}\ldots {i}_{n}}={\sum}_{t=0}^{\lfloor m/2\rfloor }{c}_{t}\{\{{\delta }_{ki}^{\otimes (m-2t)}{\delta }_{kk}^{\otimes t}{\delta }_{ii}^{\otimes t}\}\}{\delta }_{ii}^{\otimes (n-m)/2},$$

where *c*_*t*_ are known coefficients (given in Supplementary Note 1), and the double curly braces {{ }} denote symmetrization achieved by averaging over all unique permutations of the indices. Depending on the assignment of the indices, there exist multiple candidate mapping tensors for a given *n* and *m*. For example, when *n* = 3 and *m* = 1, we have ${G}_{{k}_{1}{i}_{1}{i}_{2}{i}_{3}}={c}_{0}{\delta }_{ki}{\delta }_{ii}$ (no Einstein summation on *i*), resulting in three candidates: ${G}^{1}={c}_{0}{\delta }_{{k}_{1}{i}_{1}}{\delta }_{{i}_{2}{i}_{3}},\,{G}^{2}={c}_{0}{\delta }_{{k}_{1}{i}_{2}}{\delta }_{{i}_{3}{i}_{1}},$ and ${G}^{3}={c}_{0}{\delta }_{{k}_{1}{i}_{3}}{\delta }_{{i}_{1}{i}_{2}},$ where the subscript *k*_1_*i*_1_*i*_2_*i*_3_ is dropped for clarity.

Next, identify a subset of *N*_*g*_ unique linearly independent mapping tensors from all *N* candidates **G**^1^, …, **G**^*N*^. This can be achieved via a QR factorization^43^, followed by symmetry-informed elimination according to the intrinsic symmetries of the physical tensor. For example, when *n* = 3 and *m* = 1, a QR factorization will verify that all three candidates above are linearly independent. But for a rank-3 tensor with symmetry ${T}_{{i}_{1}{i}_{2}{i}_{3}}={T}_{{i}_{1}{i}_{3}{i}_{2}}$, **G**^2^ and **G**^3^ yield identical outcomes upon contraction with **T**; thus, only **G**^1^ and **G**^2^ are retained as unique mapping tensors.

Finally, construct the decomposition projector **H** and embedding tensor **Q** to map between a physical tensor with arbitrary rank and symmetry and its natural representation:

6 $${{{{\bf{H}}}}}_{m+n}^{p}	={\sum}_{q=1}^{N}{h}_{pq}{{{{\bf{G}}}}}_{m+n}^{q}\hfill\\ {{{{\bf{Q}}}}}_{m+n}^{p}	={{{{\bf{G}}}}}_{m+n}^{p}+{\sum}_{q={N}_{g}+1}^{N}{\beta }_{qp}{{{{\bf{G}}}}}_{m+n}^{q},$$

where *p* = 1, 2, …, *N*_*g*_, and *h*_*pq*_ and *β*_*qp*_ (expressions given in Supplementary Note 1) are coefficients that can be determined by enforcing the orthonormal condition between different **G**^*q*^. With them, one can utilize ${{{{\bf{X}}}}}_{m}^{p}={{{{\bf{H}}}}}_{m+n}^{p}{\odot }^{n}{{{{\bf{T}}}}}_{n}$ to extract the natural tensors from an ordinary tensor, and, conversely, utilize

7 $${{{{\bf{T}}}}}_{n}={\sum}_{m=0}^{n}{\sum}_{p=1}^{{N}_{g}}{{{{\bf{Q}}}}}_{m+n}^{p}{\odot }^{m}{{{{\bf{X}}}}}_{m}^{p}$$

to reconstruct an ordinary tensor from its natural representations.

In summary, our theoretical contributions are twofold. First, we have developed a systematic approach to decomposing and reconstructing physical tensors of arbitrary rank and symmetry using natural tensors, which is not available in the literature (to our knowledge). Second, we have derived explicit expressions for the projection tensors (Eqs. (2), (4), and (6)) that consist of only Kronecker deltas and Levi–Civita symbols, which can be precomputed and stored, enabling efficient natural tensor operations.

### The CarNet model

**Fig. 2:**
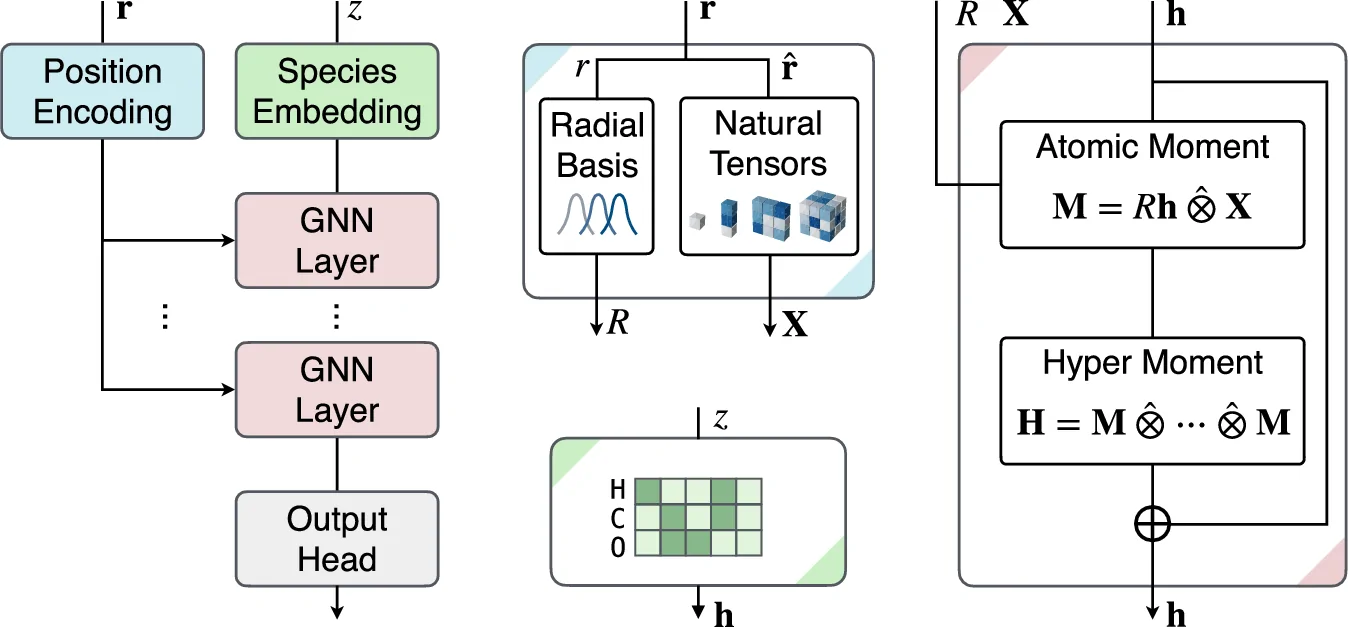
Overview of the CarNet model architecture. The relative distance vector **r** of an atom from its neighbor is encoded using a set of radial basis functions for its magnitude *r* and natural tensors constructed from polyadics of the unit vector $\hat{{{{\bf{r}}}}}$. The atomic species *z* is encoded using a learnable embedding to generate the initial atom features **h**. With the radial part *R*, the angular part **X**, and the atom features **h**, each graph neural network (GNN) layer first constructs the atomic moment **M** and then the hyper moment **H** using natural tensor products (denoted by $\hat{\otimes }$). Finally, the atomic features are mapped to the target properties using an output head.

Leveraging the three fundamental operations of natural tensors and the concept of moment tensors as introduced in MTP^22^ and CAMP^25^, we propose CarNet: a theoretically grounded framework for constructing equivariant atomistic ML models. CarNet takes an atomic structure as input, generates equivariant atom features, and produces interatomic potentials or structure–property relationships, including those involving high-rank tensors. A schematic overview of the model architecture is illustrated in Fig. 2.

The model begins by encoding the atomic structure. The relative position vector **r** between an atom and its neighbor is decomposed into its scalar distance *r* and the unit directional vector $\hat{{{{\bf{r}}}}}={{{\bf{r}}}}/r$. The scalar distance *r* is then expanded into *R* using a set of radial basis functions, specifically Chebyshev polynomials of the first kind. Angular information is incorporated through $\hat{{{{\bf{r}}}}}$, which is transformed into natural tensors **X** via the first operation described in Section 2.1. Atomic numbers *z* are embedded into learnable initial atom features **h**, completing the initial encoding of the atomic structure.

The core architecture of CarNet comprises a multilayer graph neural network (GNN) that iteratively refines atom features. Within each GNN layer, the atomic moment **M** is constructed as a tensor product involving radial function *R*, current atom features **h**, and the angular component **X**. Subsequently, a self-tensor product is performed on **M**, yielding the hyper moment **H** that encodes many-body interactions^17,20,22^. Both steps employ the natural tensor product formalism introduced above, ensuring that **M** and **H** are natural tensors. Empirical results (below) indicate that typically two to three GNN layers suffice to attain high predictive accuracy.

At the final stage, relevant natural tensors derived from atom features **h** are selected to construct the desired physical quantities. For interatomic potentials, this process involves extracting the rank-0 natural tensor (scalar). For higher-rank tensorial properties, the relevant natural tensors are extracted from atom features according to their decomposition and reconstruction spectrum, and the target physical tensor is reconstructed from these components using the third operation in Section 2.1.

The overall model architecture, the reconstruction of atomic and structural physical tensors, and the training procedures are provided in the Methods section. A detailed comparison between CarNet and existing Cartesian methods for atomistic ML (e.g., TensorNet^28^, HotPP^29^, ICTP^26^, and TACE^30^) is given in Supplementary Note 2.

### Bulk LiPS and water

We first apply CarNet to develop interatomic potentials for periodic systems—specifically, inorganic lithium phosphorus sulfide (LiPS) (a solid-state electrolyte)^19^ and bulk liquid water^44^. We assess the model’s performance by computing the mean absolute error (MAE) or root mean square error (RMSE) of energies and atomic forces on the test sets, benchmarking against state-of-the-art models in the literature, including both spherical models (e.g., NequIP^19^ and MACE^20^) and Cartesian models (e.g., CACE^24^ and CAMP^25^). For both systems, CarNet configured with two GNN layers achieves the lowest reported errors, as shown in Table 1; increasing to three layers further reduces errors.

**Table 1:** Model performance on the LiPS and water datasets

Dataset	Model	Energy (meV)	Forces (meV/Å)
LiPS	NequIP^19^	0.12	7.7
	CAMP^25^	0.12	7.4
	CarNet (2 layers)	0.11	6.4
	CarNet (3 layers)	**0.09**	**5.6**
Water	BPNN^13^	2.3	120
	ACE^17^	1.7	99
	REANN^23^	0.8	53
	DeePMD^15^	2.1	92
	NequIP^19^	0.94	45
	MACE^20^	0.63	36
	CACE^24^	0.59	47
	CAMP^25^	0.59	34
	CarNet (2 layers)	**0.54**	34
	CarNet (3 layers)	**0.54**	**31**

For LiPS, mean absolute errors (MAEs) of energy and forces on the test set are reported. For water, root-mean-square errors (RMSEs) of energy and forces are reported. CAMP results are from ref. ^25^; CACE and others are from ref. ^24^.

Bold text indicates the smallest error.

Beyond low MAEs, CarNet enables stable molecular simulations to accurately predict structural and dynamical properties. While low errors in energy and forces are necessary, they are not sufficient to guarantee the physical reliability of molecular dynamics (MD) simulations; unphysical force predictions can lead to instability or drift during simulations despite good MAEs^45^.

**Fig. 3:**
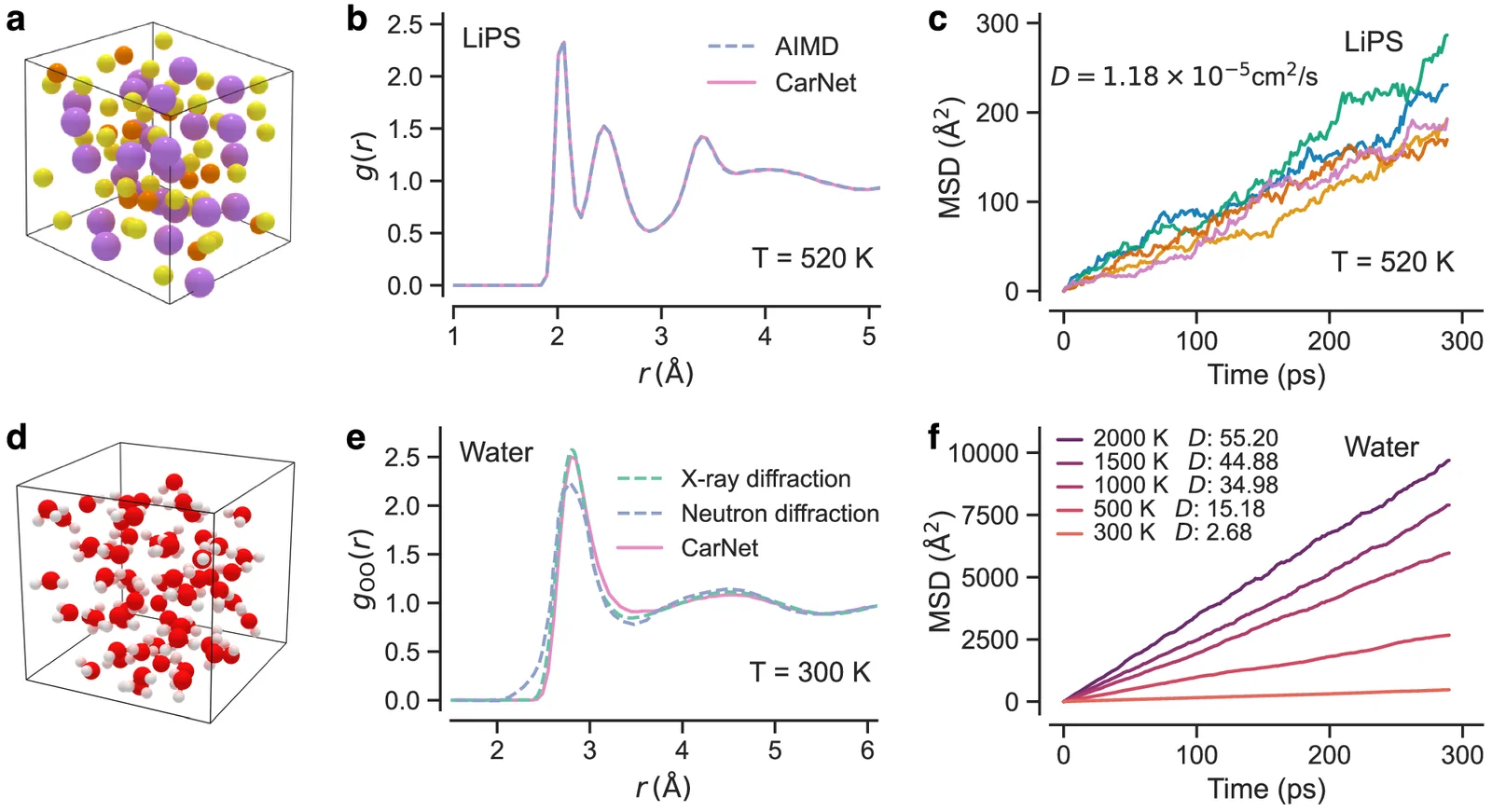
Molecular dynamics (MD) simulation results of bulk LiPS and water systems. **a**–**c** Crystal structure, radial distribution function (RDF), and mean squared displacement (MSD) of Li^+^ ions versus time for LiPS. **d**–**f** Simulation cell, RDF of oxygen-oxygen pairs, and MSD versus time for bulk water. The reference ab initio molecular dynamics (AIMD) and experimental results are at the same temperatures shown in the figures, except for the X-ray diffraction data, which is at 295 K. In panel (**c**), five MD simulations with different initial velocities were performed, and the average diffusion coefficient *D* is reported. The MD simulation for water used a 2 × 2 × 2 replication of the unit cell shown in panel (**d**). In panel (**f**), *D* is given in the units of ×10^−5^ cm^2^/s. Simulation cells are plotted using AtomViz^82^; atom colors: purple (Li), orange (P), yellow (S), red (O), and white (H). Source data are provided as a Source Data file.

To evaluate the stability and physical fidelity, we first perform MD simulations for LiPS under an NVT ensemble. For LiPS, five MD simulations with different initial velocities were carried out using a timestep of 1 fs over a total duration of 300 ps (simulation details in Methods). No instabilities or trajectory collapse were observed in any of the MD runs. We next analyze the radial distribution functions (RDF) derived from the MD trajectories. The RDF (Fig. 3b) predicted by CarNet closely matches AIMD simulation results^44^. We also compute the diffusion coefficient from the mean squared displacement (MSD) of the MD trajectories. The MSD of Li^+^ in LiPS (Fig. 3c) exhibits linear behavior, indicative of normal diffusion. The calculated diffusion coefficient from the five MD runs is *D* = (1.18 ± 0.19) × 10^−5^ cm^2^/s, consistent with AIMD estimates of *D* = 1.37 × 10^−5^ cm^2^/s^19^.

We also conducted MD simulations for water using setups similar to those for LiPS (simulation details in Methods). The RDF of oxygen-oxygen pairs in water at 300 K (Fig. 3e) agrees well with X-ray^46^ and neutron^47^ diffraction data. The predicted diffusion coefficient is *D* = 2.68 × 10^−5^ cm^2^/s at 300 K as compared with AIMD results of *D* = 2.67 × 10^−5^ cm^2^/s^44^. To further check the stability of CarNet, following^24^, we performed MD simulations at high temperatures up to 2000 K. At high temperatures, the MD simulations remained stable, and the MSD exhibited linear behavior (Fig. 3f). This demonstrates CarNet’s superior stability. We do not expect physical quantities (e.g., *D* in Fig. 3f and RDF in Supplementary Fig. 1) at high temperatures to be quantitatively accurate; this example serves only to demonstrate the stability of CarNet.

### Molecular ethanol

We also evaluated the performance of CarNet for ethanol as a small-molecule test. In addition to energies and atomic forces, the ethanol dataset^27^ includes three tensorial properties: dipole moment ***μ***, polarizability ***α***, and nuclear shielding tensor ***σ***. The dipole moment ***μ*** is a rank-1 structural tensor defined at the molecular level, representing a property of the entire molecule. The polarizability ***α*** is a rank-2 structural tensor, also defined at the molecular level. In contrast, the nuclear shielding ***σ*** is a rank-2 atomic tensor; i.e., each atom in the molecule has a shielding tensor that is sensitive to the local electronic environment. The nuclear chemical shift is a scalar property derived from ***σ*** as *δ* = Tr(***σ***)/3.

CarNet demonstrates significant improvements in predicting these properties relative to existing state-of-the-art models such as FieldSchNet^27^ and TensorNet^28^. Using a multitask learning framework with a shared backbone and multiple output heads to concurrently learn all properties, CarNet achieves the highest accuracy across all tasks (Table 2). For instance, the errors in predicting the dipole moment ***μ*** and nuclear chemical shift *δ*_all_ are ~3× smaller than those from TensorNet. Separate errors *δ*_H_, *δ*_C_, and *δ*_O_ for each element are given in Supplementary Note 3. This highlights the model’s ability to learn a unified representation for predicting distinct scalars, atomic tensors, and structural tensors. We also trained models where each property was learned individually (energy and forces jointly), observing reductions in errors across all outputs except ***α***. Models employing natural tensor representations with a maximum rank of *L* = 3 outperform their *L* = 2 counterparts, underscoring the critical role of high-rank tensors in capturing complex interatomic interactions, while highlighting limitations of models that rely on tensor representations up to rank-1 and rank-2 (e.g., FieldSchNet and TensorNet).

**Table 2:** Prediction errors in ethanol properties

	${{{\mathcal{E}}}}$ (kcal mol^−1^)	F (kcal mol^−1^ Å^−1^)	*μ* (D)	*α* (Bohr^3^)	*δ*_all_ (ppm)	*σ*_all_ (ppm)
PaiNN^80^	0.027	0.150	0.003	0.009	–	–
FieldSchNet^27^	0.017	0.128	0.004	0.008	0.169	–
TensorNet^28^	0.008	0.058	0.003	0.007	0.139	–
CarNet (multitask)	0.0063	0.034	0.0011	**0.0051**	0.044	0.065
CarNet (*L* = 2)	0.0054	0.027	0.0009	0.0076	0.037	0.057
CarNet (*L* = 3)	**0.0048**	**0.022**	**0.0007**	0.0063	**0.031**	**0.047**

Mean absolute errors (MAEs) on the test set are reported for energy ${{{\mathcal{E}}}}$, forces **F**, dipole moment ***μ***, polarizability ***α***, nuclear chemical shift *δ*_all_ and shielding tensor ***σ***_all_ for all atoms. *L* is the maximum rank of the natural tensors employed.

Bold text indicates the smallest error.

### Crystal elastic constant tensor

We now demonstrate the capability of CarNet to predict the elastic constant tensor (characterizing linear elastic response of a material under applied stress) of inorganic crystalline solids. Currently, no Cartesian atomistic ML methods can model the full (rank-4) elastic constant tensor (with up to 21 independent components depending on crystal symmetry). This is more challenging due to both the inherent complexity of the elastic constant tensor and the compositional diversity of the dataset. The dataset^6^ encompasses structures spanning all seven crystal systems, involving 84 chemical elements, thereby presenting substantial variability in symmetry, composition, and structural complexity. CarNet effectively handles this complexity, exhibiting robust applicability across diverse crystal symmetries and compositional variations.

**Fig. 4:**
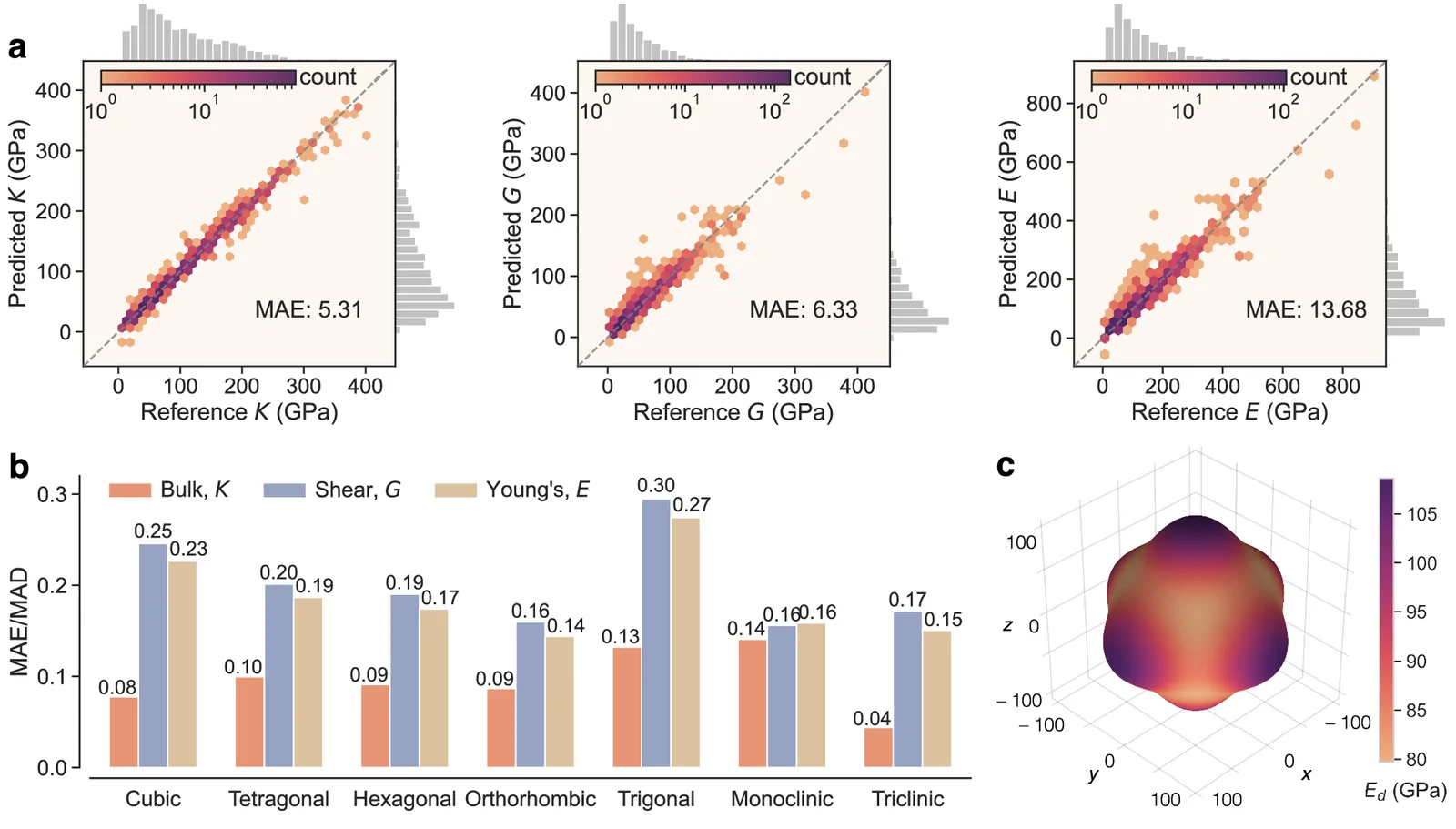
Performance of CarNet in predicting elastic properties. **a** Predicted bulk modulus *K*, shear modulus *G*, and Young’s modulus *E* compared with reference density functional theory (DFT) values. **b** Normalized error by crystal system. **c** Directional Young’s modulus *E*_*d*_ of CaS predicted by the model. The cubic symmetry of rocksalt CaS is clearly reflected in the predicted *E*_*d*_. MAE is the mean absolute error, and MAD is the mean absolute deviation. Source data are provided as a Source Data file.

CarNet directly predicts the full elastic constant tensor while inherently satisfying the two fundamental physical constraints: (1) frame indifference (coordinate system invariance) and (2) crystal symmetry adherence. Frame indifference ensures the predicted tensor is equivariant under rigid rotations of the coordinate system, while the symmetry constraint guarantees that the tensor reflects the intrinsic point-group symmetries of the crystal^6^. From the predicted rank-4 elastic constant tensor, we can obtain the Voigt stiffness matrix *C*^48^. Scalar elastic moduli such as the bulk modulus *K*, shear modulus *G*, and Young’s modulus *E* are derived from *C* using the Hill averaging scheme^49^. Quantitatively, CarNet achieves MAEs of 5.31 for *K*, 6.39 for *G*, and 13.68 for *E* over the 84 elements in the dataset (where values range from near 0 to 400 GPa for *K* and *G* and up to ~800 GPa for *E*; see Fig. 4a). The errors are ~18% lower than those by MatTen^6^ and are comparable to those by XPaiNN^50^, both of which are spherical models specifically designed for elastic constant tensors (Table 3).

**Table 3:** Prediction errors in elastic properties

	*K* (GPa)	*G* (GPa)	*E* (GPa)	*C* (GPa)
AutoMatminer^81^	9.84	9.27	22.10	–
MatSca^6^	7.32	8.63	19.87	–
MatTen^6^	7.37	8.38	20.59	4.52
XPaiNN^50^	5.40	**6.33**	14.74	–
CarNet	**5.31**	6.39	**13.68**	**3.32**

Mean absolute errors (MAEs) of the bulk (*K*), shear (*G*), and Young’s (*E*) moduli, as well as the 6 × 6 Voigt matrix (*C*) are reported. MAE of *C* is calculated component-wise.

Bold text indicates the smallest error.

A detailed analysis reveals that CarNet maintains consistent predictive accuracy across different crystal systems. The relative error, MAE normalized by the mean absolute deviation (MAD) of the reference values, is comparable among all seven crystal systems (Fig. 4b). Despite dataset imbalance (see Supplementary Fig. 2) favoring high-symmetry over low-symmetry crystals (e.g., cubic vs. triclinic), CarNet demonstrates effective transferability and generalization, indicating its ability to learn unified representations across a broad structural spectrum.

Beyond scalar moduli *K*, *G*, and *E*, the ability to predict the full elastic constant tensor enables efficient analysis of anisotropic elastic behaviors. For example, the directional Young’s modulus *E*_*d*_ can be computed for all orientations, enabling comprehensive evaluation of elastic anisotropy and directional stiffness variations (Fig. 4c). This capability underscores the utility of CarNet for materials design and mechanical property optimization where directional elastic responses are critical.

### Universal MLIP for materials

To further demonstrate the capability of CarNet to model complex systems, we train a universal MLIP for 89 chemical elements using the MatPES r^2^SCAN dataset^51^. We use the same data split and train for 100 epochs as in the original MatPES manuscript^51^. We trained three versions of CarNet all using *N*_*u*_ = 128 feature channels and a maximum natural tensor rank of *L* = 2, but with different numbers of GNN layers (1, 2, and 3) to investigate the accuracy–efficiency trade-off. The three-layer CarNet took  ~34 hours to train on a single NVIDIA RTX 5090 GPU. Compared with state-of-the-art universal MLIPs trained on this dataset, CarNet demonstrates highly competitive performance in terms of accuracy. With only a single GNN layer, it achieves test set MAEs (Table 4) comparable to those of M3GNet^52^, CHGNet^53^, and TensorNet^28^. The three-layer CarNet attains overall accuracy comparable to MACE^54^ (which, however, is additionally pretrained on the large OMat24 dataset^55^), with smaller energy and stress errors but larger force errors. UPET^56^ achieves the smallest MAEs among all models. This advantage is most plausibly attributable to two factors: UPET employs 192.89 million parameters, more than an order of magnitude larger than any other model, and, like MACE, is pretrained on OMat24 (118 million configurations) before being finetuned on MatPES. Since MACE shares the same pretraining yet performs only on par with CarNet, UPET’s additional edge appears to stem primarily from its much larger model capacity. Unlike all other models considered here, however, UPET does not guarantee rotationally invariant energy predictions, meaning that the predicted energy of a structure may change with its orientation in space. Taken together, these comparisons indicate that CarNet is highly competitive among universal MLIPs.

**Table 4:** Model performance on the MatPES r^2^SCAN test set

	Energy	Forces	Stress	Model size
	(meV/atom)	(meV/Å)	(GPa)	(millions)
M3GNet^52^	44	210	0.970	0.66
CHGNet^53^	30	156	0.735	2.70
TensorNet^28^	34	163	0.754	0.84
MACE^54^	25	84	0.711	9.06
UPET^56^	**12**	**40**	**0.201**	192.89
CarNet (1 layer)	39	175	0.897	1.60
CarNet (2 layers)	27	141	0.717	3.30
CarNet (3 layers)	23	130	0.642	7.80

Model size is measured by the number of parameters in millions. MACE and UPET are developed using data in addition to MatPES; both are pretrained on the OMat24 dataset and then finetuned on MatPES. M3GNet, CHGNet, and TensorNet results are from ref. ^51^; MACE and UPET results are evaluated by us using the model checkpoints released by the authors (see Data Availability).

Bold and underlined text indicate the smallest and second smallest errors, respectively.

**Fig. 5:**
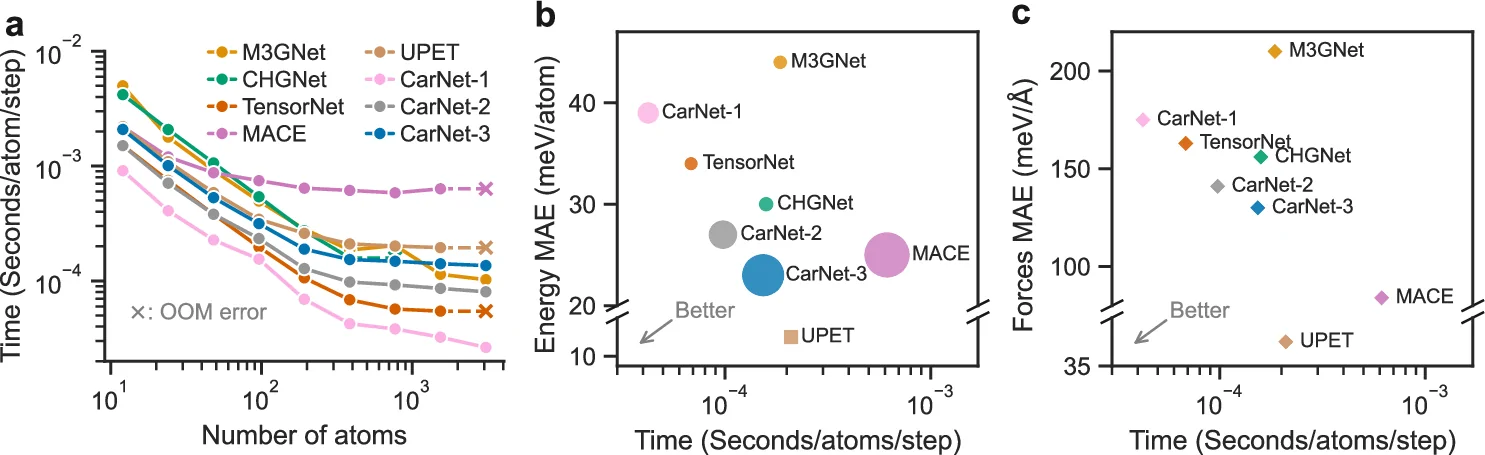
Accuracy–efficiency trade-off of various universal machine learning interatomic potentials. **a** Time per molecular dynamics (MD) step normalized by the number of atoms as a function of system size. **b** Test set mean absolute error (MAE) of energy versus time at the system size of 384 atoms. **c** Test set MAE of forces versus time. We measure the time by running MD simulations of bulk water systems with different sizes and report the average time per MD step normalized by the number of atoms. MD simulations were conducted using the Atomic Simulation Environment (ASE) on a single NVIDIA RTX 5090 GPU; the OOM label indicates out-of-memory errors during the calculations. CarNet-1, CarNet-2, and CarNet-3 denote the CarNet models with 1, 2, and 3 graph neural network (GNN) layers, respectively. In panel b, the marker size is proportional to the model size (number of parameters), except for UPET, whose model size is much larger than the others (see Table 4). Source data are provided as a Source Data file.

CarNet also demonstrates superior efficiency in terms of computational time and memory usage. We measure the inference time of the models in MD simulations of bulk water systems of different sizes. The single-layer CarNet is the fastest model of all, and the three-layer version is faster than MACE and UPET (Fig. 5a). On the NVIDIA RTX 5090 GPU with 32 GB of memory, M3GNet and all CarNet models can successfully simulate systems up to 3072 atoms (Fig. 5a), while the other models encounter out-of-memory (OOM) errors. The timing tests were also done for NaCl crystals, and similar results were obtained (see Supplementary Fig. 3). We also investigate the accuracy–efficiency trade-off by plotting the test set MAEs against the inference time for systems of 384 atoms (where all models can run without OOM errors). For both energy and forces, the CarNet models together with TensorNet and UPET form the Pareto front (Fig. 5b, c). In terms of model size (number of parameters), CarNet is an order of magnitude larger than M3GNet, CHGNet, and TensorNet, and remains slightly smaller than MACE; all are much smaller than UPET (Table 4).

## Discussion

This work develops a framework for atomistic ML by leveraging Cartesian natural tensors to systematically represent high-rank and many-body interactions in atomic structures. The proposed theory of natural tensor operations enables modeling of a wide variety of physical properties, from scalar quantities (like interatomic potential energy) to tensors of arbitrary rank and symmetry (e.g., the elastic constant tensor)—a capability unmatched by existing Cartesian approaches. Key design choices enhance computational efficiency while maintaining predictive accuracy, including the implementation of sparsified tensor product paths and the optional incorporation of atomic species dependence in model parameters.

According to the product rule^34^, the rank *l*_3_ of the natural tensor ${{{{\bf{Z}}}}}_{{l}_{3}}$ resulting from the tensor product of ${{{{\bf{X}}}}}_{{l}_{1}}$ and ${{{{\bf{Y}}}}}_{{l}_{2}}$ satisfies ∣*l*_1_ − *l*_2_∣ ≤ *l*_3_ ≤ *l*_1_ + *l*_2_. Consequently, different pairs *l*_1_, *l*_2_ can produce the same *l*_3_. For example, *l*_1_ = 1, *l*_2_ = 1 and *l*_1_ = 1, *l*_2_ = 2 can both yield *l*_3_ = 1. We define a path *p* = (*l*_1_, *l*_2_, *l*_3_) to denote such a combination. A central question pertains to the selection of paths that effectively contribute to the computation of atomic and hyper moments using Eq. (3). A straightforward approach, analogous to spherical models like NequIP^19^, involves incorporating all possible paths, which ensures maximal expressivity but incurs significant computational cost. We find that a judicious subset of paths can often achieve superior accuracy and efficiency.

**Fig. 6:**
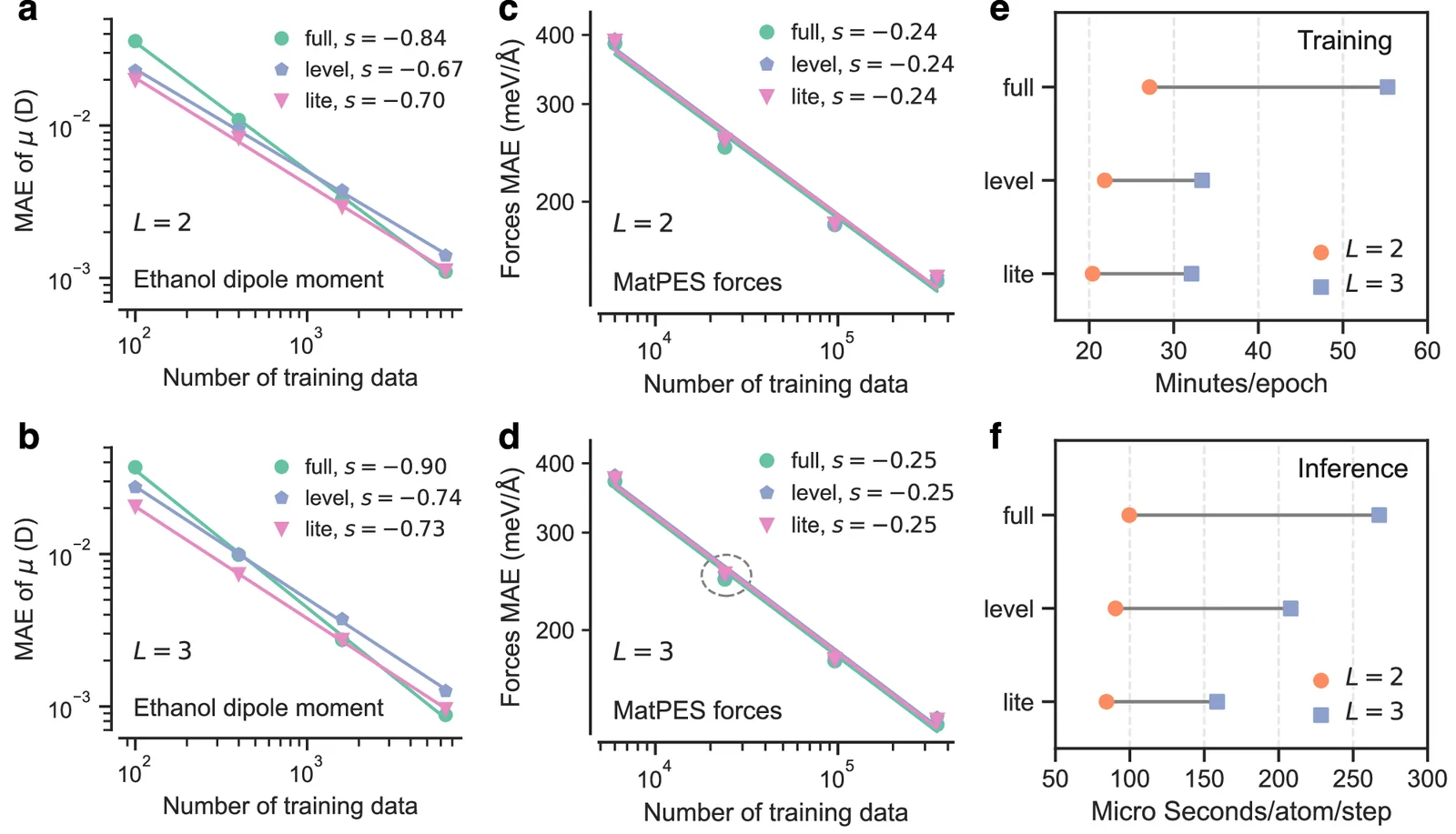
Comparison of the tensor product path selection modes. **a**, **b** Mean absolute error (MAE) on learning ethanol dipole moment ***μ*** as a function of the training data size. **c**, **d** MAE of forces on the MatPES dataset as a function of the training data size. **e**, **f** Training time on the MatPES dataset and inference time using the trained models in molecular dynamics (MD) simulations of bulk water with 384 atoms. The slope *s* is obtained by linearly fitting the learning curve in log-log space. *L* denotes the maximum rank of the natural tensors used in the model. Other hyperparameters for training the ethanol dipole moment model are: number of feature channels *N*_*u*_ = 64, number of layers *T* = 2, and cutoff radius *r*_cut_ = 5 Å. Other hyperparameters for MatPES training are: *N*_*u*_ = 128, *T* = 2, and *r*_cut_ = 5 Å. Source data are provided as a Source Data file.

We introduce three path selection modes: ‘full’, ‘lite’, and ‘level’. The full mode includes all permissible paths, whereas the lite and level modes use fewer paths by restricting the ones leading to large *l*_3_ values. Detailed descriptions and explicit tabulations of the paths are provided in Supplementary Note 4. To evaluate their performance, we trained models with these three modes on the ethanol dataset and MatPES dataset, and checked their learning curves. For ethanol dipole moment, the lite mode yields the lowest MAE when the training data size is small (Fig. 6a, b) With a larger learning curve slope *s* than the other two modes, the full mode’s MAE decreases more rapidly with increasing training data, eventually outperforming the lite mode at larger sizes. For the MatPES dataset, the full mode consistently gives the lowest force MAEs across all training data sizes (Fig. 6c, d), although the differences between the modes are less pronounced (e.g. 247, 252, and 252 meV/Å for the full, lite, and level modes, respectively, at 24,000 training configurations, circled in Fig. 6d). Additional results on using varied training settings such as different numbers of feature channels (Supplementary Fig. 4) and on other properties such as energy and stress (Supplementary Fig. 5) show the same behavior. We also compared the efficiency of the three modes, finding that the lite mode is much faster than the full mode in both model training and inference (Fig. 6d, e). For example, when using a maximum tensor rank of *L* = 2, the lite mode and the full mode take 20.4 and 27.1 minutes per epoch, respectively, to train on the MatPES dataset; they take 84 and 99 microseconds per atom per step, respectively, to run an MD simulation of bulk water with 384 atoms. For *L* = 3 with more path reduction, the efficiency advantage of the lite mode is even larger.

The different learning-curve trends can be attributed to the interplay between the expressiveness of the path selection modes and the underlying delicacy of the datasets. The full mode with all tensor product paths possesses a higher degree of expressivity that enables it to resolve the delicate interatomic interactions within the ethanol dataset. Specifically, it captures the subtle and fine details between molecular conformations along AIMD trajectories that the other two modes can overlook. As the training data size increases, this higher model capacity allows the full mode to more accurately map these delicate features, manifesting as a steeper learning-curve slope. Paradoxically, while the MatPES dataset exhibits greater chemical and structural complexity due to its inclusion of 89 elements and diverse crystal structures, it lacks the subtlety of the ethanol trajectories. Consequently, the additional degrees of freedom provided by the full mode remain underutilized on MatPES, leading to a saturation of performance where all three path selection modes result in nearly identical learning curves. To further validate this, we have conducted additional experiments on the relatively simple LiPS dataset and the more complex elasticity dataset. Exactly the same trends are observed (Supplementary Figs. 6, 7). This suggests that the effectiveness of high-rank tensor product paths is governed less by elemental diversity and more by the delicacy of the underlying potential energy surface. However, dataset complexity, particularly chemical diversity, determines the optimal choice of model parameterization. For datasets with a few chemical elements like ethanol and LiPS, we observe that making the weights in Eqs. (12) and (14) dependent on the atomic number *z*_*i*_ can enhance accuracy. This, however, does not hold for the MatPES and elasticity datasets with many chemical elements, as it leads to a large number of parameters that cannot be reliably learned from the available data.

Why Cartesian natural tensors? The field of atomistic ML has traditionally relied on spherical tensor representations and achieved considerable success. In contrast, the adoption of Cartesian representations lags behind, likely because the theoretical framework of natural tensors is less well-known and not yet fully developed. Here, we address these issues by extensions to the theory and mathematical formalism of natural tensors, as well as providing practical implementation strategies. While Cartesian and spherical representations describe the same underlying spatial reality and are mathematically connected (e.g., the tensor product $\hat{{{{\bf{r}}}}}\otimes \hat{{{{\bf{r}}}}}\otimes \cdots \otimes \hat{{{{\bf{r}}}}}$ can be expanded in terms of spherical harmonics^57^), their mathematical formulations and computational implementations differ significantly, each offering distinct advantages and limitations^26^. In addition, as discussed in Section 2.1, natural tensors possess clear physical interpretability.

There are, however, current limitations that may be further addressed to expand the potential of our approach. For example, the tensor product path selection scheme is primarily guided by empirical intuition; although different choices like the lite and level modes are comparable in the number of paths, the lite mode outperforms the level mode in general. This suggests that a more systematic approach to path selection could lead to further improvements in model performance and efficiency. Furthermore, other strategies for reducing the computational cost of the tensor product operation have also been proposed in the literature. For example, So3krates^58^ achieves efficiency by decoupling invariant features from equivariant coordinates to drive an attention mechanism; eSCN^59^ rotates frames into local coordinate systems to achieve a sparse representation; the Gaunt tensor product method^60^ maps the Clebsch–Gordan coefficients to a 2D Fourier basis, enabling the use of Fast Fourier Transforms to reduce the complexity of full tensor products. Distinct from these approaches, the high-order pair-reduced neural network^61^ avoids the tensor product completely by utilizing a hierarchical angular interaction scheme based on direct concatenation and Hadamard products. Our tensor product path selection scheme adopts a different strategy, which is to sparsify the tensor product paths. However, while these strategies were developed in the context of Clebsch–Gordan tensor products for spherical models, they may be adapted to the natural tensor product in Cartesian models. Finally, dedicated GPU kernels have been optimized for spherical tensor operations^62,63^, and similar optimizations may be implemented for natural tensor operations to further improve both training and inference efficiency.

While this work focuses on atomistic ML, the proposed Cartesian natural tensor framework is broadly applicable to other domains. Atomistic ML can be viewed as a specific instance of point cloud learning, where each point (atom) possesses attributes such as atomic number and spatial coordinates. Consequently, the natural tensor formalism and the CarNet architecture can be extended to a variety of other point cloud tasks, such as shape learning in 3D medical images^64,65^ and object recognition and segmentation in LiDAR data for autonomous driving applications^66,67^.

## Methods

### Dataset

The LiPS dataset^19^ consists of 250001 structures of lithium phosphorus sulfide (Li_6.75_P_3_S_11_) solid-state electrolyte, generated from an AIMD trajectory. Each structure consists of 27 Li atoms, 12 P atoms, and 44 S atoms. Random subsets of 1000, 1000, and 5000 structures were selected for training, validation, and test, respectively.

The water dataset^44^ consists of 1593 water configurations of 192 atoms each, obtained from AIMD simulations at 300 K. It was randomly divided into training, validation, and test sets with a split ratio of 90:5:5.

The ethanol dataset^27^ includes 10,000 molecular structures, with five target properties (energy, forces, dipole moment, polarizability, and nuclear shielding), all computed in vacuum using DFT. It was randomly divided into training, validation, and test sets with a split ratio of 8:1:1.

The MatPES r^2^SCAN dataset^51^ consists of 387,897 DFT calculations of 89 elements in various crystal structures. We use the same training, validation, and test splits as in the original work^51^, which are 80%, 10%, and 10% of the total dataset, respectively.

The elasticity dataset^25^ contains elastic constant tensors for inorganic crystals from the DFT data in the Materials Project^68^. It includes 10276 elastic constant tensors, and we use the same training, validation, and test splits as in the original work^6^, which are 80%, 10%, and 10% of the total dataset, respectively.

### Model architecture

CarNet is a graph neural network (GNN) that takes an atomic structure as input and predicts target physical properties. The model comprises four key components: atomic-species embeddings; radial and directional representations of interatomic vectors using radial basis functions and natural tensors, respectively; atomic and hyper moments constructed through natural tensor products; and output heads for predicting target properties. Below, we describe each component in detail.

An atomic structure is represented as a graph *G* = (*V*, *E*), where the nodes *V* correspond to atoms, and the edges *E* connect pairs of atoms within a cutoff radius *r*_cut_. A node *i* (atom) is characterized by three properties: the atomic coordinates **r**^*i*^, atomic number *z*_*i*_, and atom features **h**^*i*^. For each edge (*i*, *j*) we define the relative position vector as: **r**^*ij*^ = **r**^*j*^ − **r**^*i*^, which encodes the spatial relationship between atom *i* and its neighbor *j*.

The atom features **h**^*i*^ consist of a set of natural tensors, indexed by *u* and *l*. In their tensorial form, these features are represented as ${{{{\bf{h}}}}}_{ul}^{i}$, where *l* denotes the tensor rank and *u* labels the feature channels. The initial features of each atom are derived by embedding its atomic number:

8 $${{{{\bf{h}}}}}_{u0}^{i}={W}_{u{z}^{i}},$$

where ${W}_{u{z}^{i}}$ is a learnable embedding matrix. These initial atom features are scalar-valued and constitute natural tensors of rank *l* = 0.

The unit edge vector ${\hat{{{{\bf{r}}}}}}^{ij}={{{{\bf{r}}}}}^{ij}/{r}^{ij}$ with *r*^*ij*^ = ∥**r**^*ij*^∥ encodes the directional information between atoms *i* and *j*. Atom indices *i* and *j* in **r**^*ij*^ are omitted for simplicity. To capture angular interactions of various orders, we construct the polyadic tensor ${{{{\bf{U}}}}}_{l}=\hat{{{{\bf{r}}}}}\otimes \hat{{{{\bf{r}}}}}\otimes \cdots \otimes \hat{{{{\bf{r}}}}}$ where the tensor product is taken *l* times, producing a rank-*l* tensor. Each **U**_*l*_ can be decomposed into its natural tensor components, resulting in the set:

9 $${{{{\bf{X}}}}}_{0}=1,\,{{{{\bf{X}}}}}_{1}=\left[\begin{array}{c}{\hat{r}}_{x}\\ {\hat{r}}_{y}\\ {\hat{r}}_{z}\\ \end{array}\right],\,{{{{\bf{X}}}}}_{2}=\left[\begin{array}{ccc}{\hat{r}}_{x}^{2}-{\sigma }_{0}&{\hat{r}}_{x}{\hat{r}}_{y}&{\hat{r}}_{x}{\hat{r}}_{z}\\ {\hat{r}}_{y}{\hat{r}}_{x}&{\hat{r}}_{y}^{2}-{\sigma }_{0}&{\hat{r}}_{y}{\hat{r}}_{z}\\ {\hat{r}}_{z}{\hat{r}}_{x}&{\hat{r}}_{z}{\hat{r}}_{y}&{\hat{r}}_{z}^{2}-{\sigma }_{0}\end{array}\right]$$

and so forth, where ${\hat{r}}_{x}$, ${\hat{r}}_{y}$ and ${\hat{r}}_{z}$ are the Cartesian components of $\hat{{{{\bf{r}}}}}$, and ${\sigma }_{0}=({\hat{r}}_{x}^{2}+{\hat{r}}_{y}^{2}+{\hat{r}}_{z}^{2})/3$, which is one-third the trace of **U**_2_—ensuring **X**_2_ is traceless (more explicitly, ${{{{\bf{X}}}}}_{l}={{{{\bf{X}}}}}_{l}^{ij}$).

The interatomic distance (edge length) *r*^*ij*^ is expanded in a set of radial basis functions *B*_*u*_ indexed by channel *u*. Basis functions are constructed as linear combinations of Chebyshev polynomials of the first kind *Q*_*β*_,

10 $${B}_{u}(r)={\sum}_{\beta=0}^{{N}_{\beta }}{W}_{u\beta }{Q}_{\beta }\left(\frac{r}{{r}_{{{{\rm{cut}}}}}}\right){f}_{c}\left(\frac{r}{{r}_{{{{\rm{cut}}}}}}\right),$$

where *N*_*β*_ is the maximum degree of the Chebyshev polynomials and *W*_*uβ*_ are learnable weights. The radial expansion is similar to that used in MTP^69^ and CAMP^25^, but here the weights *W*_*uβ*_ are shared across different atomic species, significantly reducing the total number of learnable parameters; this is advantageous for datasets with many chemical elements.

Using the atom features, angular information, and radial basis, we construct an atomic moment that encodes the local environment of each atom:

11 $${{{{\bf{M}}}}}_{u{l}_{3},p}^{i}=\frac{1}{\sqrt{| {{{\mathcal{N}}}}| }}{\sum}_{j\in {{{{\mathcal{N}}}}}_{i}}{R}_{u{l}_{3}{l}_{1}{l}_{2}}{{{{\bf{h}}}}}_{u{l}_{1}}^{j}\hat{\otimes }{{{{\bf{X}}}}}_{{l}_{2}}^{ij},$$

where ${{{{\mathcal{N}}}}}_{i}$ is the set of neighboring atoms within a distance of *r*_cut_ of atom *i*, $| {{{\mathcal{N}}}}|$ is the average number of neighbors per atom in the training set, ${R}_{u{l}_{3}{l}_{1}{l}_{2}}$ is a learnable radial function, and $\hat{\otimes }$ denotes the natural tensor product (as defined in Section 2.1). The radial function ${R}_{u{l}_{3}{l}_{1}{l}_{2}}$ is obtained by passing the radial basis *B*_*u*_ through a multilayer perceptron (MLP): ${R}_{u{l}_{3}{l}_{1}{l}_{2}}=\,{\mbox{MLP}}\,({B}_{u})$ using two hidden layers with the SiLU nonlinearity^70^. Different MLPs are used for each combination of indices (*l*_3_, *l*_2_, *l*_1_). Similar to spherical tensor products, the product of natural tensors of ranks *l*_1_ and *l*_2_ can produce a natural tensor of rank *l*_3_. The triplet *p* = (*l*_1_, *l*_2_, *l*_3_) is called a path and determines the tensorial combination in Eq. (11).

Atomic moment tensors of the same rank but originating from different tensorial paths are linearly combined as follows:

12 $${{{{\bf{M}}}}}_{ul}^{i}={\sum}_{{u}^{{\prime} }p}{W}_{u{u}^{{\prime} }l,p}^{{z}_{i}}{{{{\bf{M}}}}}_{{u}^{{\prime} }l,p}^{i},$$

where ${W}_{u{u}^{{\prime} }l,p}^{{z}_{i}}$ are trainable weights. To reduce the number of parameters, these weights are factored as ${W}_{u{u}^{{\prime} }l,p}^{{z}_{i}}={W}_{u{u}^{{\prime} }l}^{{z}_{i}}{W}_{p}$.

From the atomic moments, we construct the hyper moment,

13 $${{{{\bf{H}}}}}_{ul}^{i}={{{{\bf{M}}}}}_{u{l}_{1}}^{i}\hat{\otimes }{{{{\bf{M}}}}}_{u{l}_{2}}^{i}\hat{\otimes }\cdots \hat{\otimes }{{{{\bf{M}}}}}_{u{l}_{v}}^{i}\quad (v\,{{{\rm{of}}}}\,{{{\bf{M}}}}).$$

where $\hat{\otimes }$ denotes the natural tensor product and *v* is the number of atomic moments being combined. An atomic moment encodes two-body interactions between an atom and its neighbors. By taking tensor product of atomic moments with themselves, higher-order interactions are incorporated: three-body, four-body, and beyond. Specifically, a hyper moment of degree *v* captures interactions up to body order *v* + 1. The hyper moment thus provides a systematic and complete description of the local atomic environment^22,57^, which is essential for constructing systematically improvable interatomic potentials. This approach is analogous to the B-basis used in ACE^17^ and MACE^20^. Similar to atomic moments, multiple tensor paths can generate hyper moments of the same rank *l* that may be linearly combined. An efficient algorithm for evaluating Eq. (13) iteratively is provided in Supplementary Note 5.

Atom features are then updated using a residual connection^71^ by combining the hyper moments with atom features of the previous layer:

14 $${{{{\bf{h}}}}}_{ul}^{i,t}={{{{\bf{H}}}}}_{ul}^{i}+{\sum}_{{u}^{{\prime} }}{W}_{u{u}^{{\prime} }l}^{{z}_{i}}{{{{\bf{h}}}}}_{{u}^{{\prime} }l}^{i,t-1},$$

where *t* is the layer index.

The feature update process is performed for *N*_layer_ layers, producing a sequence of atom features: ${{{{\bf{h}}}}}_{ul}^{i,1}$, ${{{{\bf{h}}}}}_{ul}^{i,2}$, … , ${{{{\bf{h}}}}}_{ul}^{i,{N}_{{{{\rm{layer}}}}}}$. Depending on the modeling target, the features from either all layers or a subset of layers are used to construct the final output. Empirically, two or three layers are sufficient for interatomic potentials, atomic tensors, and structure tensors.

For interatomic potentials, the atomic energy *E*^*i*^ is derived from *l* = 0 scalar atom features ${{{{\bf{h}}}}}_{u0}^{i,t}$ across all layers

15 $${E}^{i}={\sum}_{t=1}^{{N}_{{{{\rm{layer}}}}}}V({{{{\bf{h}}}}}_{u0}^{i,t}),$$

in which *V* is implemented as an MLP using two hidden layers with the SiLU nonlinearity for the last layer where *t* = *N*_layer_, and a linear function, $V({{{{\bf{h}}}}}_{u0}^{i,t})={\sum }_{u}{W}_{u}^{t}{{{{\bf{h}}}}}_{u0}^{i,t}$, for earlier layers (*t* < *N*_layer_). The total potential energy is the sum over all atoms:

16 $$E={\sum}_{i}\sigma {E}^{i}+{\mu }_{{z}_{i}},$$

where ${\mu }_{{z}_{i}}$ is the atomic energy for species *z*_*i*_ (obtained directly from DFT calculations or computed via a linear fit to the total energies of the training set), and *σ* is the root mean square of the atomic forces computed on the training set^54,72^. Forces are obtained via the negative gradient: ${{{{\bf{F}}}}}_{i}=-\frac{\partial E}{\partial {{{{\bf{r}}}}}_{i}}.$

For atomic tensors, the atom features ${{{{\bf{h}}}}}_{ul}^{i,1}$, ${{{{\bf{h}}}}}_{ul}^{i,2}$, … , ${{{{\bf{h}}}}}_{ul}^{i,{N}_{{{{\rm{layer}}}}}}$ from all layers are linearly combined along the channel dimension *u* to produce a rank-*l* natural tensor for each atom:

17 $${{{{\bf{v}}}}}_{l}^{i}={\sum}_{t,u}{W}_{u}^{t}{{{{\bf{h}}}}}_{ul}^{i,t}.$$

Relevant natural tensors are then selected to reconstruct physical tensors.

For example, the rank-0 ${{{{\bf{v}}}}}_{0}^{i}$, rank-1 ${{{{\bf{v}}}}}_{1}^{i}$ and rank-2 ${{{{\bf{v}}}}}_{2}^{i}$ natural tensors are used to reconstruct the rank-2 nuclear shielding tensor ***σ*** for each atom *i*. For the shielding tensor, there is only one unique independent mapping tensor for each rank, i.e., *N*_*g*_ = 1 for all *m* in Eq. (7). Therefore, the reconstruction of the shielding tensor is given by:

18 $${{{{\boldsymbol{\sigma }}}}}^{i}={\sum}_{m=0}^{2}{{{{\bf{Q}}}}}_{m+2}{\odot }^{m}{{{{\bf{X}}}}}_{m}$$

where ${{{{\bf{X}}}}}_{0}={{{{\bf{v}}}}}_{0}^{i}$, ${{{{\bf{X}}}}}_{1}={{{{\bf{v}}}}}_{1}^{i}$, and ${{{{\bf{X}}}}}_{2}={{{{\bf{v}}}}}_{2}^{i}$ are the natural tensors of rank 0, 1, and 2, respectively.

For structural tensors, natural tensors of different ranks *l* are first generated for each atom according to Eq. (17). They are then aggregated across all atoms by taking the average or sum, depending on whether the target physical tensor is intensive or extensive. Then relevant natural tensors are selected to reconstruct the physical tensor.

For extensive quantities (e.g., the dipole moment **u** and polarizability ***α***), the tensors are summed:

19 $${{{{\bf{X}}}}}_{l}={\sum}_{i}{{{{\bf{v}}}}}_{l}^{i}.$$

According to the decomposition and recombination spectrum (given in Supplementary Table 3), the dipole moment **u** only needs the rank-1 natural tensor **X**_1_ for its reconstruction. It can be obtained via Eq. (7) as:

20 $${{{\bf{u}}}}={{{{\bf{Q}}}}}_{m+1}{\odot }^{m}{{{{\bf{X}}}}}_{m},$$

where *m* = 1. The polarizability ***α*** is a symmetric rank-2 tensor, which can be reconstructed from the rank-0 and rank-2 natural tensors (**X**_0_ and **X**_2_) as:

21 $${{{\boldsymbol{\alpha }}}}={\sum}_{m\in \{0,2\}}{{{{\bf{Q}}}}}_{m+2}{\odot }^{m}{{{{\bf{X}}}}}_{m}.$$

For the intensive elastic constant tensor **C**, the average is taken:

22 $${{{{\bf{X}}}}}_{l}=\frac{1}{N}{\sum}_{i}{{{{\bf{v}}}}}_{l}^{i},$$

where *N* is the total number of atoms in the structure. The rank-4 elastic constant tensor **C** is more complex: it decomposes into two rank-0 natural tensors (${{{{\bf{X}}}}}_{0}^{1}$ and ${{{{\bf{X}}}}}_{0}^{2}$), two rank-2 natural tensors (${{{{\bf{X}}}}}_{2}^{1}$ and ${{{{\bf{X}}}}}_{2}^{2}$), and a rank-4 natural tensor **X**_4_ (see Supplementary Table 3). The two tensors ${{{{\bf{X}}}}}_{0}^{1}$ and ${{{{\bf{X}}}}}_{0}^{2}$ of the same rank are obtained by employing separate ${W}_{u}^{t}$ in Eq. (17); similarly for ${{{{\bf{X}}}}}_{2}^{1}$ and ${{{{\bf{X}}}}}_{2}^{2}$. Then the elastic constant tensor is reconstructed via Eq. (7) as:

23 $${{{\bf{C}}}}={\sum}_{m\in \{0,2,4\}}{\sum}_{g=1}^{{N}_{g}(m)}{{{{\bf{Q}}}}}_{m+4}^{g}{\odot }^{m}{{{{\bf{X}}}}}_{m}^{g},$$

where *N*_*g*_(0) = 2, *N*_*g*_(2) = 2, and *N*_*g*_(4) = 1.

### Normalization

Properly normalizing the internal features of a neural network is critical for ensuring stable and efficient training. In this work, we adopt the default uniform initialization scheme in PyTorch^73^ for all learnable weights, but we update the initialization bounds so that all network components yield outputs with approximately zero mean and unit variance. Furthermore, when summing over the features of neighboring atoms to compute the atomic moment in Eq. (11), we normalize the sum by dividing by the square root of the average number of neighbors, $\sqrt{| {{{\mathcal{N}}}}| }$, in the training set. This normalization assumes that contributions from different terms are uncorrelated, such that their variances are additive^72^.

Normalizing learning targets is equally crucial for model performance^72^. This normalization process is equivalent to the choice of scale and shift parameters for the model’s output. For interatomic potentials, we use the root mean square of the atomic forces *σ* as the scale parameter and the atomic energy ${\mu }_{{z}_{i}}$ as the shift parameter, as described in Eq. (16). For tensorial properties, we perform normalization in the natural tensor space rather than the Cartesian physical tensor space, using rank-dependent scale and shift parameters. For scalar natural tensors (*l* = 0), the shift *μ*_*l*_ and scale *σ*_*l*_ are set to the mean and standard deviation of the target scalar values across the training set. For higher-rank natural tensors (*l* > 0), the shift is set to zero (*μ*_*l*_ = 0) to maintain equivariance, while the scale *σ*_*l*_ is set to the root mean square of the Frobenius norm of the target natural tensors of the same rank. Applying these parameters to the atomic natural tensors, Eq. (17) becomes: ${{{{\bf{v}}}}}_{l}^{i}={\sigma }_{l}\left({\sum }_{t,u}{W}_{u}^{t}{{{{\bf{h}}}}}_{ul}^{i,t}\right)+{\mu }_{l}$. We note that although this normalization is conducted at the atomic level, the formulation remains valid for structural targets, which are simply linear combinations of atomic values. For example, under this normalization, Eq. (22) becomes: ${{{{\bf{v}}}}}_{l}=\frac{1}{N}{\sum }_{i}\left({\sigma }_{l}{{{{\bf{v}}}}}_{l}^{i}+{\mu }_{l}\right)={\sigma }_{l}\left(\frac{1}{N}{\sum }_{i}{{{{\bf{v}}}}}_{l}^{i}\right)+{\mu }_{l},$ which is identical to normalizing the structural tensor directly.

### Model training

Interatomic potentials are trained by minimizing a loss function of energy and forces (and stress if available). For a given atomic structure, the loss is

24 $$l(\theta )={w}_{{{{\mathcal{E}}}}}{\left(\frac{{{{\mathcal{E}}}}-\hat{{{{\mathcal{E}}}}}}{N}\right)}^{2}+{w}_{{{{\rm{F}}}}}\frac{{\sum }_{i=1}^{N}\parallel {{{{\bf{F}}}}}_{i}-\hat{{{{{\bf{F}}}}}_{i}}{\parallel }^{2}}{3N}+{w}_{{{{\rm{S}}}}}\parallel {{{\bf{S}}}}-\hat{{{{\bf{S}}}}}{\parallel }^{2},$$

where *N* is the number of atoms, ${{{\mathcal{E}}}}$, **F**_*i*_, and **S** are the model-predicted energy, forces, and stress, $\hat{{{{\mathcal{E}}}}}$, $\hat{{{{{\bf{F}}}}}_{i}}$, and $\hat{{{{\bf{S}}}}}$ are the corresponding reference values, and ${w}_{{{{\mathcal{E}}}}}$, *w*_F_, and *w*_S_ are the energy, force, and stress weighting factors.

For atomic tensor properties (e.g., nuclear shielding tensor), the loss is computed as

25 $$l(\theta )=\frac{1}{N}\mathop{\sum}_{i=1}^{N}\parallel {{{{\boldsymbol{\sigma }}}}}^{i}-{\hat{{{{\boldsymbol{\sigma }}}}}}^{i}{\parallel }^{2},$$

where ***σ***^*i*^ is the predicted Cartesian rank-2 nuclear shielding tensor for atom *i*, and ${\hat{{{{\boldsymbol{\sigma }}}}}}^{i}$ is its reference value. For structural tensor properties (e.g., dipole moment, polarizability, and elastic constant tensors), the loss per structure is

26 $$l(\theta )=\parallel {{{\bf{T}}}}-\hat{{{{\bf{T}}}}}{\parallel }^{2},$$

where **T** and $\hat{{{{\bf{T}}}}}$ are the predicted and reference tensors. The total loss to minimize at each optimization step is the mean of the losses of a minibatch of configurations.

For the elastic constant tensor dataset and the MatPES dataset, we also used the Huber loss^74^ for training, which is less sensitive to outliers than the mean squared error loss. It is defined as

27 $${l}_{\delta }=\left\{\begin{array}{ll}\frac{1}{2}\parallel y-\hat{y}{\parallel }^{2},\hfill\quad &\,{\mbox{for}}\,\parallel y-\hat{y}\parallel < \delta \hfill\\ \delta \parallel y-\hat{y}\parallel -\frac{1}{2}{\delta }^{2},\quad &\,{\mbox{otherwise}}\,\hfill\end{array}\right.,$$

where *y* and $\hat{y}$ denote the predicted and reference values, respectively (e.g., **T** and $\hat{{{{\bf{T}}}}}$ for the elastic constant tensor), and *δ* is a hyperparameter that determines the threshold for switching between the quadratic and linear loss regimes.

The models are implemented in PyTorch^73^ and trained using PyTorch Lightning^75^. Optimization is performed with the AdamW optimizer^76^. A cosine annealing learning rate schedule is employed, starting with an initial learning rate depending on the specific task. During model performance evaluation, an exponential moving average of the model weights is maintained, with decay rates of 0.999 for interatomic potential models and 0.99 for tensor property models. Hyperparameters are selected based on model performance on the validation set, and all results correspond to the test set.

Detailed model training hyperparameters for all tasks are provided in Supplementary Table 1.

### Molecular dynamics

MD simulations are performed in the NVT ensemble using the Nosé–Hoover thermostat. For LiPS, the simulation employs a cell size identical to that used in the training data, consisting of 83 atoms. The system is maintained at 520 K with a timestep of 1 fs and a Nosé–Hoover chain damping time of 20 fs. The total simulation duration is 300 ps. The thermostat is implemented with the ASE NoseHooverChain^77^, using a single chain. Simulations for water are similarly conducted using a cell with 512 molecules at temperatures from 300 to 2000 K.

The diffusion coefficient *D* is calculated from the mean squared displacement (MSD) using the Einstein relation^78^:

28 $$D={{\lim }_{t\to \infty }}\frac{\left\langle \frac{1}{N}{\sum}_{i}^{N}{\left\vert {{{{\bf{r}}}}}_{i}(t)-{{{{\bf{r}}}}}_{i}(0)\right\vert }^{2}\right\rangle }{2nt},$$

where *N* is the number of diffusing atoms (lithium for LiPS, oxygen for water), **r**_*i*_(*t*) is the position of atom *i* at time *t*, 〈 ⋅ 〉 denotes an ensemble average over multiple time origins or trajectories, and *n* = 3 indicates diffusion in three dimensions. In practice, the diffusion coefficient is computed from the slope of a linear fit to the MSD versus 2*nt*, as implemented in ASE^77^. The data from the initial 10 ps is discarded; the remaining data is used in the fitting (Fig. 3c, f). This ensures an accurate estimation of the diffusion coefficient from the long-time linear regime of the MSD.

### Reporting summary

Further information on research design is available in the Nature Portfolio Reporting Summary linked to this article.

## Supplementary information

Supplementary Information

Reporting Summary

Transparent Peer Review file

## Source data

Source Data

## Data Availability

Source data are provided with this paper. All datasets used to train models are publicly available: The LiPS dataset is at https://archive.materialscloud.org/record/2022.45, the water dataset is at https://doi.org/10.1073/pnas.1815117116, the ethanol dataset is at http://quantum-machine.org, the elasticity dataset is at https://doi.org/10.5281/zenodo.8190849, and the MatPES dataset (v2025.1) is at https://matpes.ai. The CarNet models generated in this study have been deposited in a GitHub repository at https://github.com/wengroup/carnet_run. The MACE and UPET models trained on the OMAT dataset and finetuned on the MatPES dataset are publicly available. The ‘MACE-matpes-r2scan-omat-ft.model’ checkpoint is at https://github.com/ACEsuit/mace-foundations/releases/tag/mace_matpes_0, and the ‘pet-omatpes-l-v0.1.0.ckpt’ checkpoint is at https://huggingface.co/lab-cosmo/upet/tree/main/models. Source data are provided with this paper.

## Code availability

The CarNet source code is at https://github.com/wengroup/carnet; the version used to generate the results reported in this work, commit 186071c81b49cd1d254c751ed4a4f6b8f1b85df9, is archived at Zenodo^79^. The code for natural tensor operations is at https://github.com/wengroup/natt. Scripts for training models, running MD simulations, and analyzing the results are at https://github.com/wengroup/carnet_run.
