# Supplementary material for: Atomistic machine learning with irreducible Cartesian natural tensors
Source: Nat Commun. 2026 Aug 27;17:10282. doi: 10.1038/s41467-026-77263-z (PMC13619602; doi:10.1038/s41467-026-77263-z)
Supplement: Supplementary file 1 [file 41467_2026_77263_MOESM1_ESM.pdf]

Supplementary Information: Atomistic Machine Learning with Irreducible Cartesian Natural Tensors

Qun Chen,[1] A. S. L. Subrahmanyam Pattamatta,[2]
Boyu Wang,[1] David J. Srolovitz,[2] and Mingjian Wen[1,*]

[1] *Institute of Fundamental and Frontier Sciences, University of Electronic Science and Technology of China, Chengdu, China*

[2] *Department of Mechanical Engineering, The University of Hong Kong, Hong Kong SAR, China*

* mjwen@uestc.edu.cn

CONTENTS

Supplementary Note 1. Cartesian Natural Tensor Theory 10

Supplementary Note 2. Comparison with other Cartesian methods 23

Supplementary Note 3. Nuclear chemical shifts 25

Supplementary Note 4. Tensor product modes 26

Supplementary Note 5. Algorithm to compute hyper moment 30

LIST OF FIGURES

1 Water RDF at different temperatures . . . 3
2 Elastic tensor dataset by crystal system . . . 3
3 Inference time for NaCl systems . . . 4
4 Ethanol dipole moment learning curve . . . 5
5 MatPES learning curve . . . 6
6 LiPS learning curve . . . 7
7 Elastic tensor learning curve . . . 8

LIST OF TABLES

1 Hyperparameters and training details . . . 9
2 Decomposition spectrum of Cartesian tensors . . . 17
3 Decomposition spectrum of physical tensors . . . 20
4 Nuclear shielding of ethanol . . . 25
5 Tensor product paths for $L = 2$ . . . 27
6 Tensor product paths for $L = 3$ . . . 28
7 Tensor product paths for $L = 4$ . . . 29

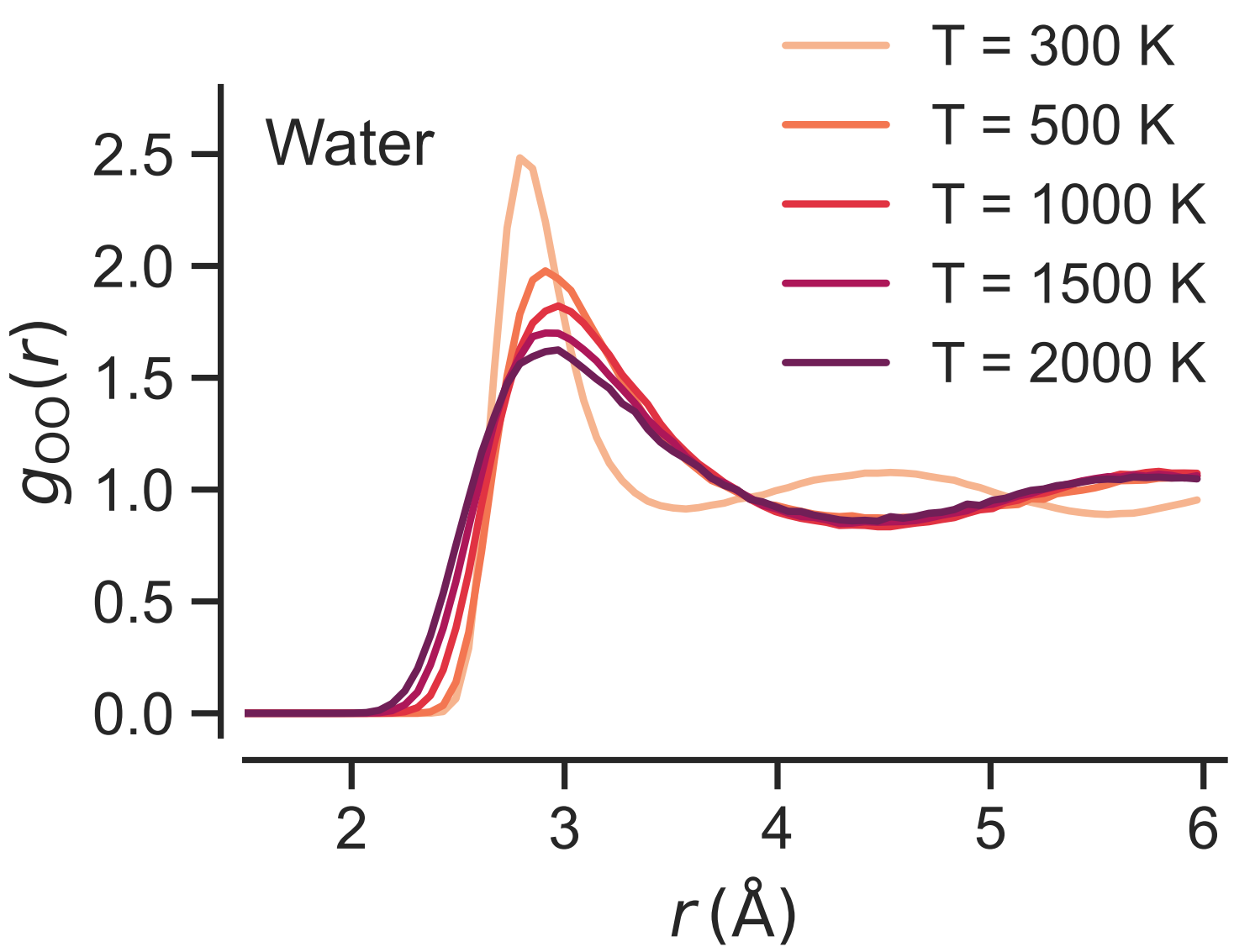

Supplementary Figure 1: Radial distribution function (RDF) of water computed from the molecular dynamics (MD) simulations using the CarNet model at different temperatures. Source data are provided as a Source Data file.

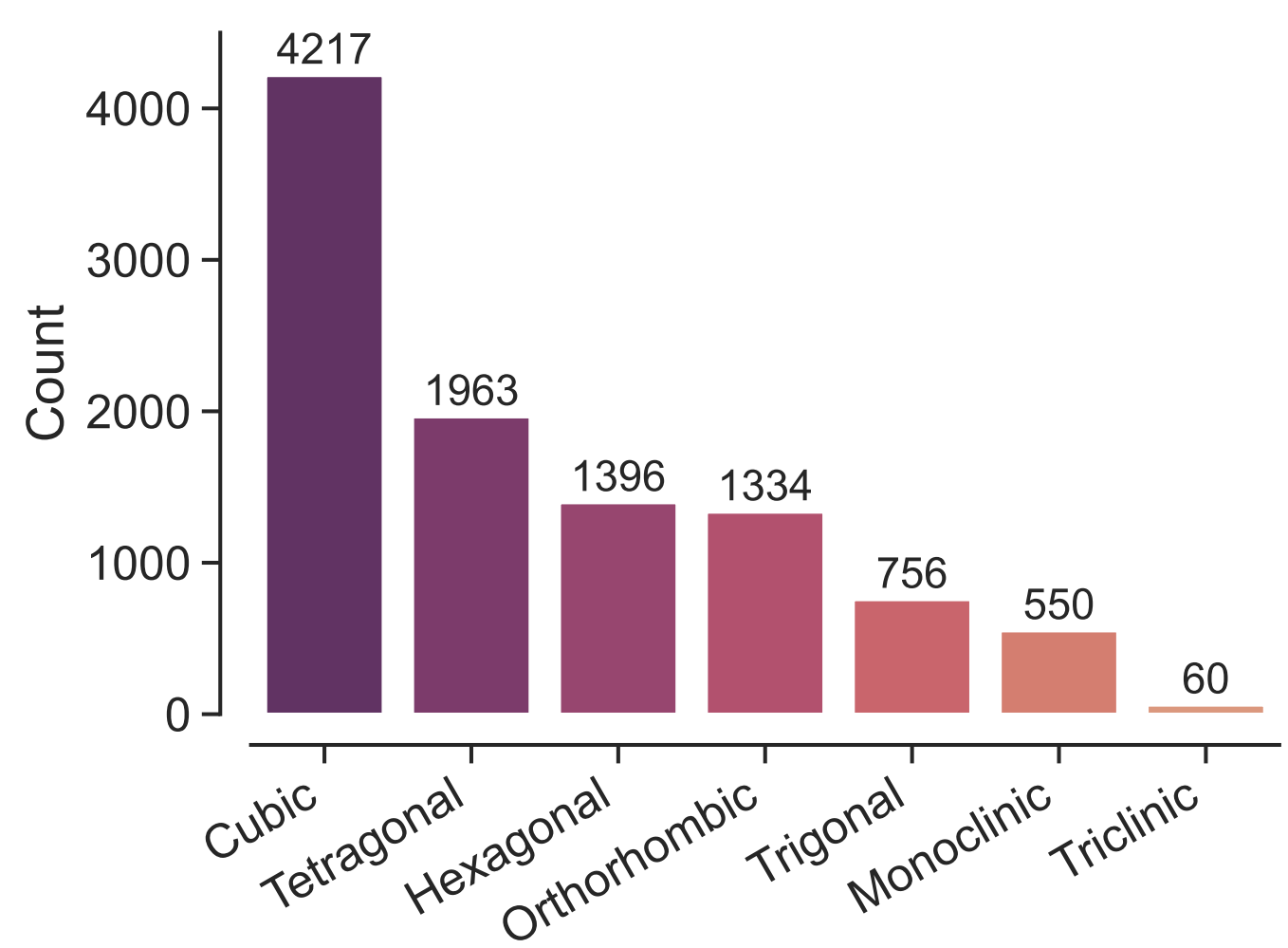

Supplementary Figure 2: Histogram of the elastic tensor dataset categorized by crystal system, showing the number of structures (y-axis) for each crystal symmetry (x-axis). Source data are provided as a Source Data file.

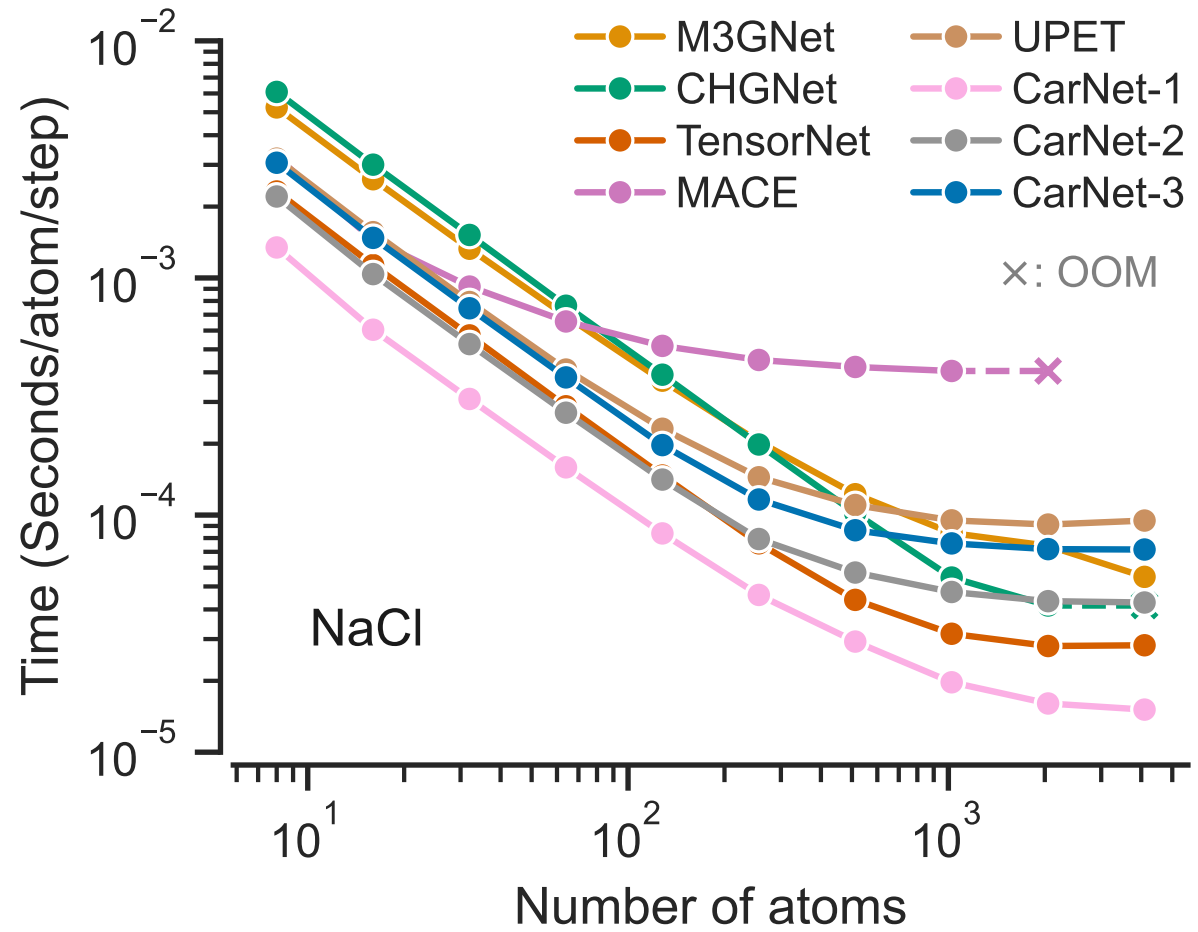

Supplementary Figure 3: Inference time of various universal machine learning interatomic potentials (MLIPs) for NaCl systems with different model size. Out-of-memory (OOM) occurs for some models when the system size is large. Source data are provided as a Source Data file.

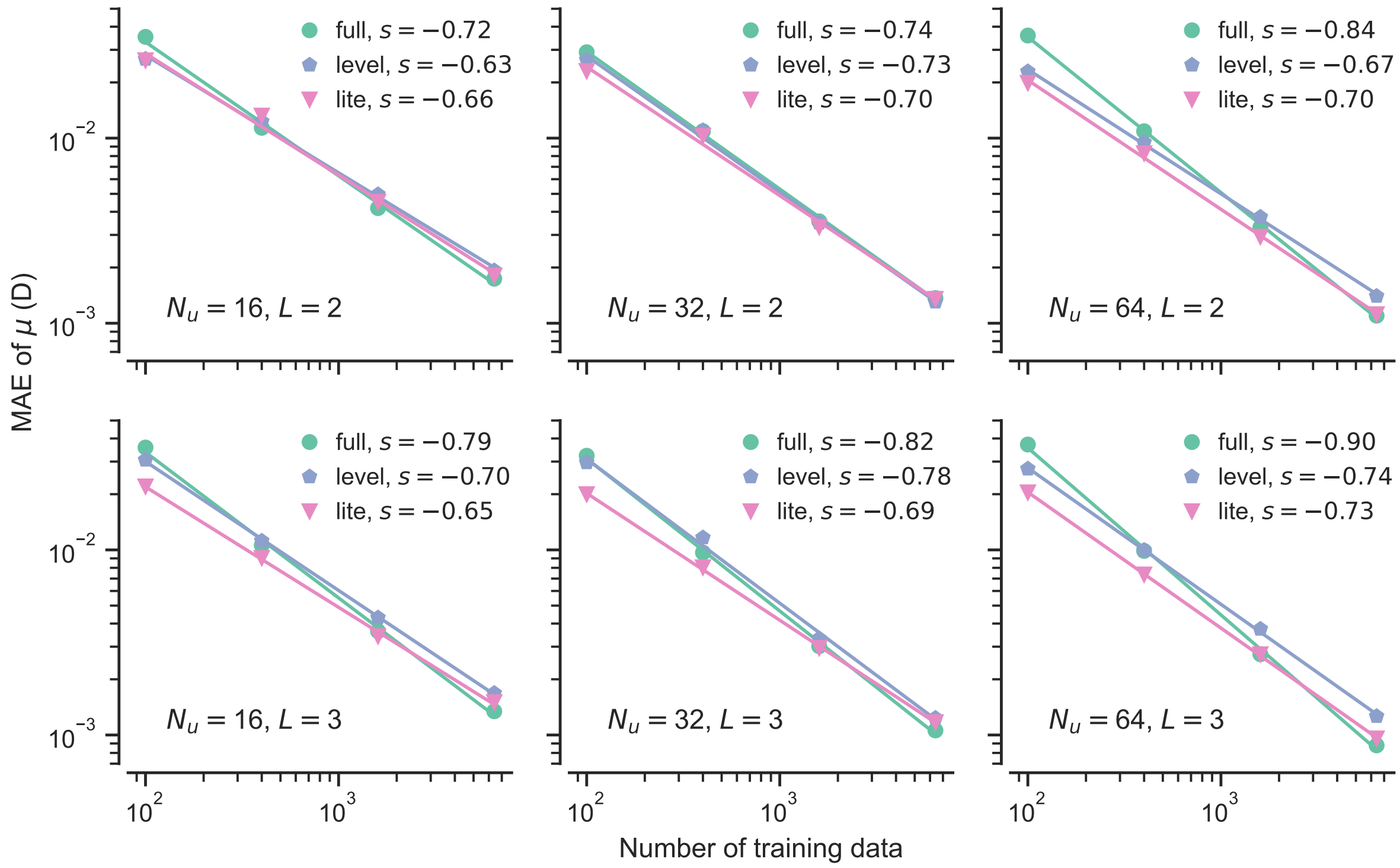

Supplementary Figure 4: Learning curve of CarNet evaluated on ethanol dipole moment μ. The mean absolute error (MAE) is computed on the test set and plotted against the number of data samples used to train the model. N_u is the number of feature channels, and L is the maximum allowed rank of the natural tensors used in the model. Other model training hyperparameters are: number of layers $T = 2$, correlation degree $v = 3$, and cutoff radius $r_{\mathrm{cut}} = 5$ Å. Source data are provided as a Source Data file.

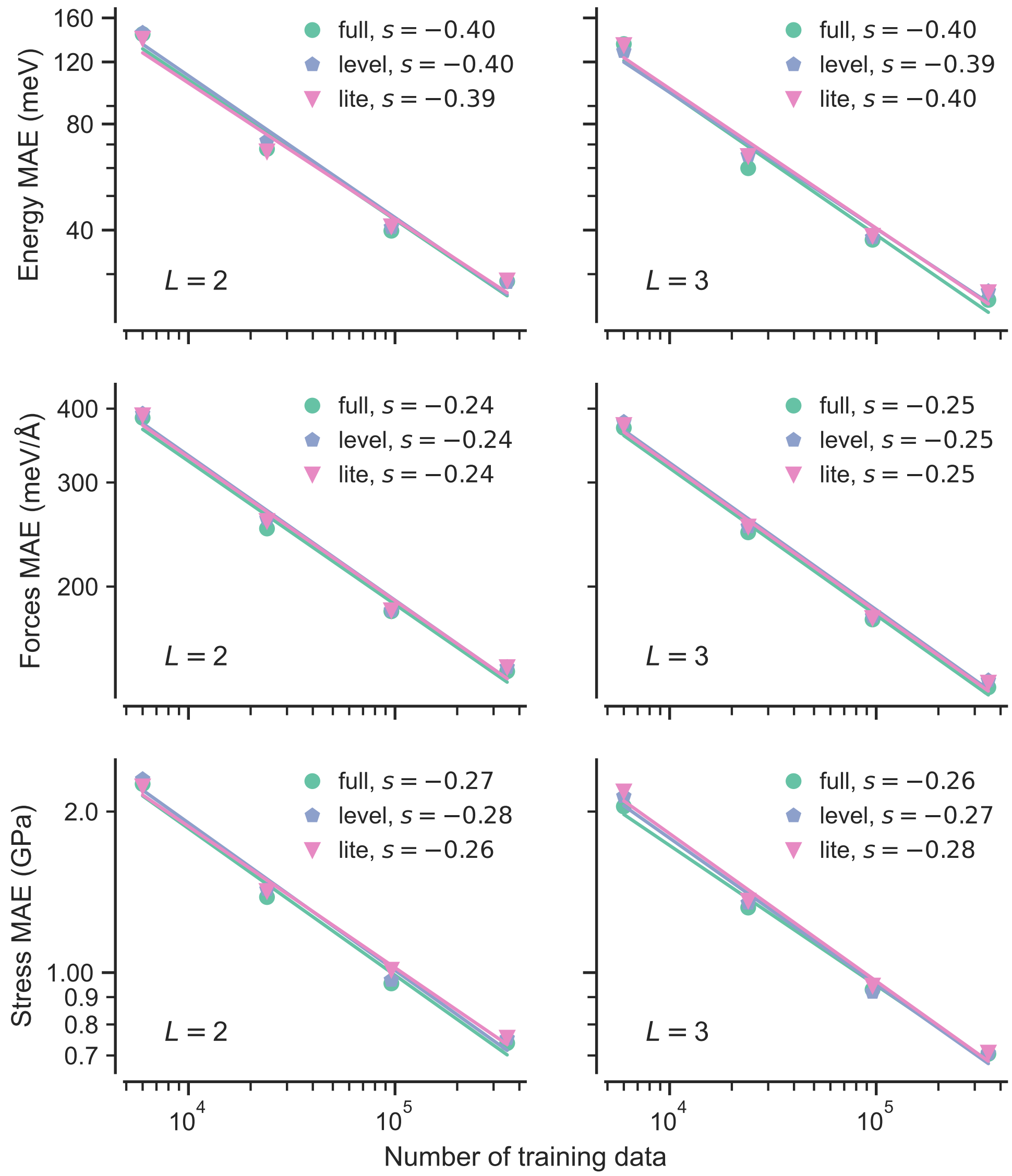

Supplementary Figure 5: Learning curve of CarNet evaluated on the MatPES dataset. The mean absolute error (MAE) is computed on the test set and plotted against the number of data samples used to train the model. L denotes the maximum allowed rank of the natural tensors used in the model. Other model training hyperparameters are: number of feature channels $N_u = 128$, number of layers $T = 2$, correlation degree $v = 3$, and cutoff radius $r_{\text{cut}} = 5$ Å. Source data are provided as a Source Data file.

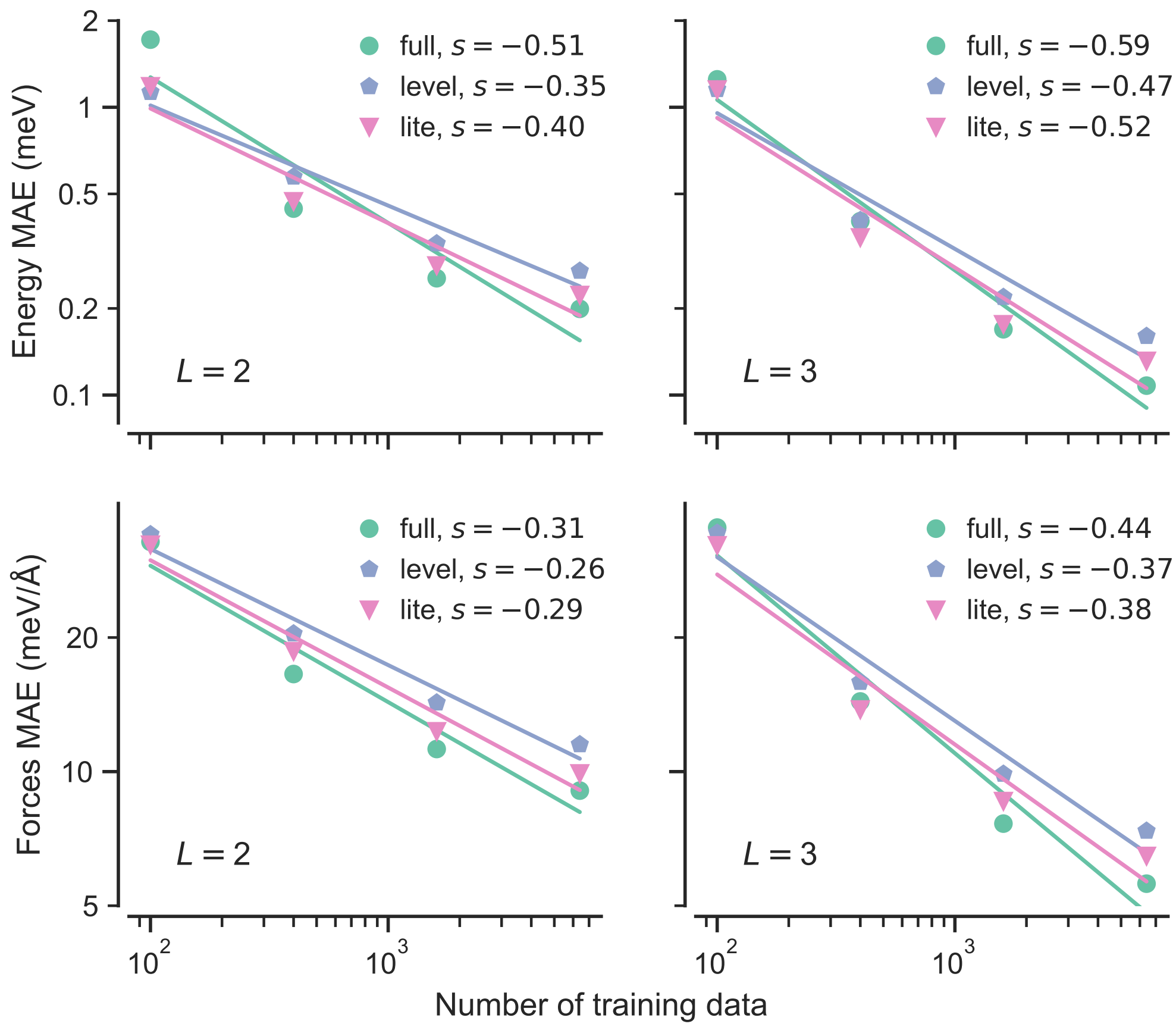

Supplementary Figure 6: Learning curve of CarNet evaluated on the LiPS dataset. The mean absolute error (MAE) is computed on the test set and plotted against the number of data samples used to train the model. L denotes the maximum allowed rank of the natural tensors used in the model. Other model training hyperparameters are: number of feature channels $N_u = 32$, number of layers $T = 2$, correlation degree $v = 3$, and cutoff radius $r_{\text{cut}} = 5$ Å. Source data are provided as a Source Data file.

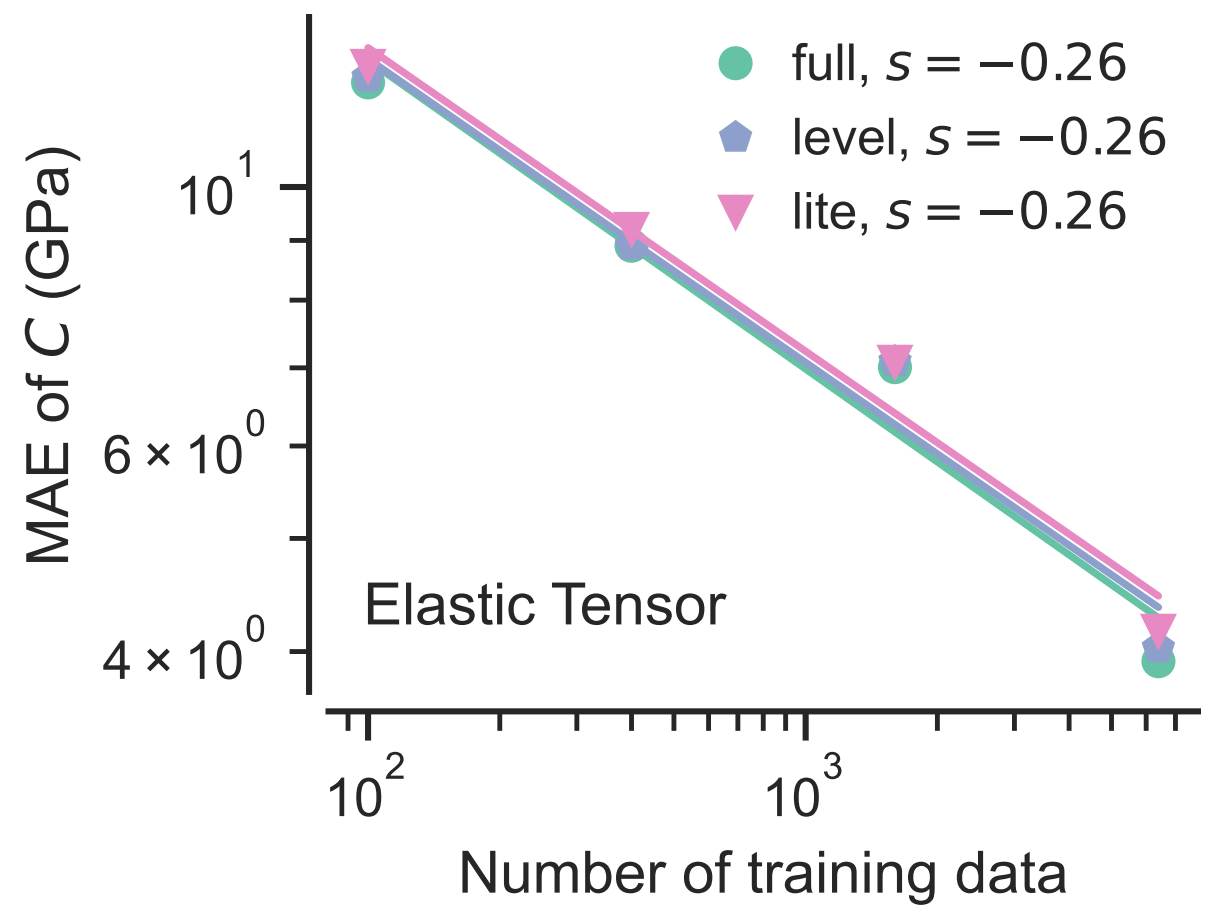

Supplementary Figure 7: Learning curve of CarNet evaluated on the elastic tensor dataset. The mean absolute error (MAE) is computed on 6×6 Voigt components of the elastic tensor on the test set. Hyperparameters used in model training are: maximum rank of natural tensors $L = 4$, number of feature channels $N_u = 48$, number of layers $T = 2$, correlation degree $v = 3$, and cutoff radius $r_{\text{cut}} = 5$ Å. Source data are provided as a Source Data file.

Supplementary Table 1: Hyperparameters and training Details. 'Weight mode' determines the type of weights used in Eqs. 12 and 14 in the main text. Mode 1 (default): The weights depend on the atomic number z, denoted as $W^{z_i}_{uu'l,p}$ and $W^{z_i}_{uu'l}$. Mode 2: The weights do not depend on the atomic number z, becoming $W_{uu'l,p}$ and $W_{uu'l}$

Dataset	LiPS	Water	Ethanol $(\mathcal{E}, \mathbf{F})$	Ethanol $(\boldsymbol{\mu}, \boldsymbol{\alpha}, \boldsymbol{\sigma})$	Ethanol (multitask)	Elasticity	MatPES
TP mode	full	lite	full	full	full	lite	lite
Weight mode	1	1	1	1	1	2	2
Max rank L	3	2	3	3	3	4	2
Corr. degree v	3	2	3	3	3	3	3
# layers T	2/3	2/3	2/3	3	3	3	2/3
# channels N_u	48	48	64	64	128	64	128
# Chebyshev basis	8	8	8	8	8	8	8
Cutoff r_{cut} (Å)	6	5	5	5	5	6	6
Dataset size	[a]	1593	10000	10000	10000	10276	387897
Data split		random	random	random	random	using [1]	using [2]
Split ratio		9:0.5:0.5	8:1:1	8:1:1	8:1:1	8:1:1	8:1:1
Max epochs	2000	2000	2000	2000	2000	1000	100
Learning rate	0.002	0.001	0.002	0.002	0.002	0.001	0.0002
Batch size	4	4	20	20	20	20	32
EMA decay	0.999	0.999	0.99	0.99	0.99	0.99	0.999
Weight decay	1×10^{-8}	1×10^{-8}	1×10^{-8}	1×10^{-8}	1×10^{-8}	1×10^{-1}	0
Loss function	MSE	MSE	MSE	MSE	MSE[b]	Huber(10)[c]	Huber (0.01)
Weight $w_{\mathcal{E}}$	1	10	1	-	1	-	1
Weight w_{F}	1	1	1	-	1	-	1
Weight w_{S}	-	-	-	-	-	-	10

[a] The LiPS dataset consists of 250001 configurations from an AIMD trajectory. Subsets of 1000, 1000, and 5000 are randomly sampled for training, validation, and testing, respectively.
[b] Weights for $\boldsymbol{\mu}$, $\boldsymbol{\alpha}$, $\boldsymbol{\sigma}$ set to 1.
[c] Value in the parentheses is the Huber δ.

Supplementary Note 1. Cartesian Natural Tensor Theory

In the main text, we have provided a concise summary of the key formulas of the Cartesian natural tensor theory that we have developed and employed in this work. Here, we present a detailed discussion of the theory. We also provide a computer program to evaluate the formulas presented here, giving both symbolic and numerical results: https://github.com/wengroup/carnet.

Let's first define some notation.

- A bold uppercase letter with a subscript n denotes a rank-n tensor (e.g., $\mathbf{T}_n, \mathbf{U}_n, \mathbf{X}_n$). When the rank is not important for the context, we use a bold uppercase letter without a subscript to denote the tensor (e.g. $\mathbf{T}, \mathbf{U}, \mathbf{X}$).

- A normal uppercase letter with multiple subscripts denotes the components of a tensor in indicial notation (e.g., $T_{i_1 i_2 \dots i_n}, U_{i_1 i_2 \dots i_n}, X_{i_1 i_2 \dots i_n}$).

- $\otimes$ denotes the tensor product between two tensors. For example, for a rank-m tensor $\mathbf{A}_m$ and a rank-n tensor $\mathbf{B}_n$, their tensor product $\mathbf{C}_{m+n} = \mathbf{A}_m \otimes \mathbf{B}_n$ is a rank-$(m+n)$ tensor with components $C_{i_1 i_2 \dots i_m j_1 j_2 \dots j_n} = A_{i_1 i_2 \dots i_m} B_{j_1 j_2 \dots j_n}$.

- $\mathbf{A}^{\otimes k}$ denotes the k-fold tensor product of a tensor $\mathbf{A}$ with itself, i.e., $\mathbf{A}^{\otimes k} = \mathbf{A} \otimes \mathbf{A} \otimes \cdots \otimes \mathbf{A}$ (k of $\mathbf{A}$).

- $\odot^k$ denotes the rank-k contraction between two tensors. For example, for a rank-m tensor $\mathbf{A}_m$ and a rank-n tensor $\mathbf{B}_n$ ($m \geq n$), their rank-k contraction $\mathbf{C}_{m+n-2k} = \mathbf{A}_m \odot^k \mathbf{B}_n$ is a rank-$(m+n-2k)$ tensor with components $C_{i_1 i_2 \dots i_{m-k} j_1 j_2 \dots j_{n-k}} = A_{i_1 i_2 \dots i_{m-k} p_1 p_2 \dots p_k} B_{j_1 j_2 \dots j_{n-k} p_1 p_2 \dots p_k}$, where repeated indices ($p_1, p_2, \dots, p_k$, here) imply summation over them according to the Einstein summation convention.

1.1. Natural tensors from a unit vector

For a unit vector $\hat{\mathbf{r}}$, a natural tensor of rank n from it can be created by first constructing a rank-n symmetric polyadic tensor $\mathbf{U} = \hat{\mathbf{r}}^{\otimes n} = \hat{\mathbf{r}} \otimes \cdots \otimes \hat{\mathbf{r}}$ (n of $\hat{\mathbf{r}}$ in the tensor product),

and then removing the traces to get the rank-n symmetric traceless natural tensor $\mathbf{V}_n$ [3]:

$$V_{i_1\ldots i_n} = C\sum_{t=0}^{\lfloor n/2\rfloor}(-1)^t\frac{(2n-2t-1)!!}{(2n-1)!!}\left\{\delta_{i_1i_2}\ldots\delta_{i_{2t-1}i_{2t}}U_{i_{2t+1}\ldots i_n\underbrace{ppqq\ldots}_{t\text{ pairs}}}\right\} \tag{1}$$

where $C = \frac{(2n-1)!!}{n!}$ is a normalization factor (explained below), $\lfloor x\rfloor$ denotes the largest integer not greater than x, the curly braces $\{\}$ denote full symmetrization achieved by summing over all unique permutations, each term in $\{\}$ contains t Kronecker deltas, and t pairs of indices from $\mathbf{U}$ are contracted (repeated indices $p, q, \ldots$ are summed, following the Einstein summation convention). The rank-0 and rank-1 natural tensors are 1 (scalar) and $\hat{\mathbf{r}}$ (vector), respectively. Eq. (1) can also be written as [4]

$$V_{i_1\ldots i_n} = C\sum_{t=0}^{\lfloor n/2\rfloor}(-1)^t\frac{(2n-2t-1)!!}{(2n-1)!!}\{\delta_{i_1i_2}\ldots\delta_{i_{2t-1}i_{2t}}\hat{r}_{2t+1}\hat{r}_{2t+2}\ldots\hat{r}_n\}, \tag{2}$$

given that $\hat{\mathbf{r}}$ is a unit vector.

The normalization constant $C = \frac{(2n-1)!!}{n!}$ is chosen such that the rank-n contraction with an arbitrary unit vector $\hat{\mathbf{b}}$ gives the Legendre polynomial of degree n:

$$\mathbf{V}_n \odot^n \hat{\mathbf{b}}^{\otimes n} = P_n(\hat{\mathbf{r}}\cdot\hat{\mathbf{b}}). \tag{3}$$

If $\hat{\mathbf{b}}$ is chosen to be $\hat{\mathbf{r}}$, then it normalizes to 1, i.e.,

$$\mathbf{V}_n \odot^n \hat{\mathbf{r}}^{\otimes n} = 1. \tag{4}$$

The normalization factor is optional but convenient in practice. In our computer implementation, we provide an option to include or exclude C. In the main text, we have omitted the normalization factor for simplicity.

Neither Eq. (1) nor Eq. (2) is efficient for numerical evaluation due to the summing over different t and the full symmetrization. **We propose an efficient method to obtain natural tensors from a unit vector.** Eq. (1) can be rewriten as

$$\mathbf{V}_n = \mathbf{H}_{2n}\odot^n \mathbf{U}_n \qquad V_{k_1\ldots k_n} = H_{k_1\ldots k_n i_1\ldots i_n}U_{i_1\ldots i_n}, \tag{5}$$

where

$$H_{k_1\ldots k_n i_1\ldots i_n} = C\sum_{t=0}^{\lfloor n/2\rfloor}(-1)^t\frac{(2n-2t-1)!!}{(2n-1)!!}\{\delta_{ki}^{\otimes n-2t}\delta_{kk}^{\otimes t}\}\delta_{ii}^{\otimes t}, \tag{6}$$

in which the superscript $^{\otimes t}$ is a shorthand notation for the t-fold tensor product of Kronecker deltas (e.g., $\delta_{ki}^{\otimes 2} = \delta_{k_1 i_1}\delta_{k_2 i_2}$ and $\delta_{ii}^{\otimes 2} = \delta_{i_1 i_2}\delta_{i_3 i_4}$), and the curly braces $\{\,\}$ denote symmetrization achieved by summing over all unique permutations of the indices.

This is equivalent to a rearrangement of the summation and symmetrization in Eq. (1). However, it can be much more efficient for numerical evaluation, since one can precompute $\mathbf{H}$ (both symbolically and numerically) for different n and store them, and then $\mathbf{V}_n$ can be efficiently evaluated by performing a single rank-n contraction between $\mathbf{H}_{2n}$ and $\mathbf{U}_n$.

Here, $\mathbf{H}_{2n}$ plays the same role as the spherical harmonic functions to construct spherical tensors from unit vectors. Note that Eq. (6) applies only to completely symmetric tensors such as $\mathbf{U}$; a generalization to arbitrary symmetries is given in "Decomposition and reconstruction of physical tensors".

Let's give some concrete examples of $\mathbf{H}_{2n}$ up to $n = 3$. As discussed above, the rank-0 and rank-1 natural tensors from a unit vector $\hat{\mathbf{r}}$ are 1 and $\hat{\mathbf{r}}$, respectively, so we have

$$\begin{aligned} &H_0 = r_{i_1} \quad \text{for } n = 0 \\ &H_{k_1 i_1} = \delta_{k_1 i_1} \quad \text{for } n = 1 \end{aligned} . \tag{7}$$

For $n = 2$, Eq. (6) is

$$\begin{aligned} H_{k_1 k_2 i_1 i_2} &= C \sum_{t=0}^{1} (-1)^t \frac{(4-2t-1)!!}{(3)!!} \{\delta_{ki}^{\otimes 2-2t} \delta_{kk}^{\otimes t}\} \delta_{ii}^{\otimes t} \\ &= C\left(\frac{3!!}{3!!}\{\delta_{ki}^2\} - \frac{1!!}{3!!}\{\delta_{kk}\}\delta_{ii}\right) \\ &= C\left(\{\delta_{k_1 i_1}\delta_{k_2 i_2}\} - \frac{1}{3}\{\delta_{k_1 k_2}\}\delta_{i_1 i_2}\right) \\ &= C\left(\delta_{k_1 i_1}\delta_{k_2 i_2} - \frac{1}{3}\delta_{k_1 k_2}\delta_{i_1 i_2}\right). \end{aligned} \tag{8}$$

We note that $\{\delta_{k_1 i_1}\delta_{k_2 i_2}\} = \delta_{k_1 i_1}\delta_{k_2 i_2}$ instead of $\delta_{k_1 i_1}\delta_{k_2 i_2} + \delta_{k_1 i_2}\delta_{k_2 i_1}$, because:

- The symmetrization $\{\}$ should be performed only over **unique** permutations.
- $U_{i_1 i_2}$ is symmetric, i.e., the two indices i_1 and i_2 are interchangeable;

For $n = 3$, Eq. (6) evaluates to

$$H_{k_1 k_2 k_3 i_1 i_2 i_3} = C\left(\delta_{k_1 i_1}\delta_{k_2 i_2}\delta_{k_3 i_3} - \frac{1}{5}\delta_{k_1 i_1}\delta_{k_2 k_3}\delta_{i_2 i_3} - \frac{1}{5}\delta_{k_1 i_2}\delta_{k_2 k_3}\delta_{i_1 i_3} - \frac{1}{5}\delta_{k_1 i_3}\delta_{k_2 k_3}\delta_{i_1 i_2}\right). \tag{9}$$

1.2. Product of natural tensors

The tensor product between two natural tensors can be expressed as a direct sum of a set of natural tensors [4, 5]. Given natural tensors $\mathbf{X}_{l_1}$ of rank l_1 and $\mathbf{Y}_{l_2}$ of rank l_2, their product

$$\mathbf{Z}_{l_3} = \mathbf{X}_{l_1} \hat{\otimes} \mathbf{Y}_{l_2} \tag{10}$$

is a tensor whose rank lies in the range $|l_1 - l_2| \leq l_3 \leq l_1 + l_2$ (analogous to spherical tensors [6]), where $\hat{\otimes}$ represents the natural tensor product. For example, when $l_1 = 1$ and $l_2 = 2$, $\mathbf{Z}_{l_3}$ is of rank 1, 2, or 3. The tensor product can be obtained as follows [4].

For an even $l_1 + l_2 - l_3 = 2d$,

$$\mathbf{Z}_{l_3} = \mathbf{X}_{l_1} \hat{\otimes} \mathbf{Y}_{l_2} = C_{l_1 l_2 l_3} \sum_{t=0}^{\min(l_1,l_2)-d} (-1)^t 2^t \frac{(2l_3 - 2t - 1)!!}{(2l_3 - 1)!!} \{\mathbf{X}_{l_1} \odot^{d+t} \mathbf{Y}_{l_2} \otimes \mathbf{I}^{\otimes t}\} \tag{11}$$

For an odd $l_1 + l_2 - l_3 = 2d + 1$,

$$\mathbf{Z}_{l_3} = \mathbf{X}_{l_1} \hat{\otimes} \mathbf{Y}_{l_2} = D_{l_1 l_2 l_3} \sum_{t=0}^{\min(l_1,l_2)-d-1} (-1)^t 2^t \frac{(2l_3 - 2t - 1)!!}{(2l_3 - 1)!!} \{\boldsymbol{\epsilon} : \mathbf{X}_{l_1} \odot^{d+t} \mathbf{Y}_{l_2} \otimes \mathbf{I}^{\otimes t}\}, \tag{12}$$

where the curly braces { } denote full symmetrization achieved by summing over all unique permutations, $\boldsymbol{\epsilon}$ is the Levi-Civita symbol, and the double colon operator : denotes a double contraction, which is performed between $\boldsymbol{\epsilon}$ and one index of $\mathbf{X}_{l_1}$ and one index of $\mathbf{Y}_{l_2}$. The normalization factors are given by [4]

$$C_{l_1 l_2 l_3} = \frac{l_1! l_2! (2l_3 - 1)!! ((J_1 + 1)/2) ((J_2 + 1)/2)!}{l_3! J_1!! J_2!! J_3!! (J/2)!} \tag{13}$$

and

$$D_{l_1 l_2 l_3} = \frac{2 l_1! l_2! (2l_3 - 1)!! (J_1/2)! (J_2/2)!}{(l_3 - 1)! (J_1 + 1)!! (J_2 + 1)!! (J_3 + 1)!! ((J + 1)/2)!}, \tag{14}$$

where $J = l_1 + l_2 + l_3$ and $J_i = J - 2l_i - 1$. The normalization factor $C_{l_1 l_2 l_3}$ is chosen such that when $\mathbf{X}_{l_1}$ and $\mathbf{Y}_{l_2}$ are constructed from a unit vector $\hat{\mathbf{r}}$ as in Eq. (1), that is

$$\mathbf{X}_{l_1} = \mathbf{V}_{l_1} \quad \text{and} \quad \mathbf{Y}_{l_2} = \mathbf{V}_{l_2}, \tag{15}$$

$\mathbf{Z}_{l_3}$ reduces to $\mathbf{V}_{l_3}$ constructed from the same unit vector $\hat{\mathbf{r}}$. In this case, according to Eqs (3) and (4), we have

$$\mathbf{Z}_{l_3} \odot^{l_3} \hat{\mathbf{b}}^{\otimes l_3} = P_{l_3}(\hat{\mathbf{r}} \cdot \hat{\mathbf{b}}), \tag{16}$$

and

$$\mathbf{Z}_{l_3} \odot^{l_3} \hat{\mathbf{r}}^{\otimes l_3} = 1. \tag{17}$$

The normalization factor $D_{l_1 l_2 l_3}$ is chosen such that when $\mathbf{X}_{l_1}$ is constructed from a unit vector $\hat{\mathbf{a}}$ and $\mathbf{Y}_{l_2}$ is constructed from a unit vector $\hat{\mathbf{b}}$ as $\hat{\mathbf{b}}$ approaches $\hat{\mathbf{a}}$, we have

$$\lim_{\hat{\mathbf{b}}\to\hat{\mathbf{a}}} \frac{|\mathbf{Z}_{l_3} \odot^{l_3-1} \hat{\mathbf{a}}^{\otimes l_3-1}|}{|\hat{\mathbf{a}} \times \hat{\mathbf{b}}|} = 1, \tag{18}$$

where $|\cdot|$ denotes the norm of a vector.

Eqs. Eq. (11) and Eq. (12) are not efficient for numerical evaluation due to the summing over different t and the full symmetrization. **We propose an efficient evaluation method as follows.** They can be rewritten as

$$\mathbf{Z}_{l_3} = \mathbf{H}_{l_1+l_2+l_3} \odot^{l_1} \mathbf{X}_{l_1} \odot^{l_2} \mathbf{Y}_{l_2} \qquad Z_{k_1\ldots k_{l_3}} = H_{k_1\ldots k_{l_3} i_1\ldots i_{l_1} j_1\ldots j_{l_2}} X_{i_1\ldots i_{l_1}} Y_{j_1\ldots j_{l_2}}. \tag{19}$$

For even $l_1 + l_2 - l_3 = 2d$:

$$H_{k_1\ldots k_{l_3} i_1\ldots i_{l_1} j_1\ldots j_{l_2}} = C_{l_1 l_2 l_3} \sum_{t=0}^{\min(l_1,l_2)-d} (-2)^t \frac{(2l_3-2t-1)!!}{(2l_3-1)!!} \{\delta_{ik}^{\otimes l_1-(d+t)} \delta_{jk}^{\otimes l_2-(d+t)} \delta_{kk}^{\otimes t}\} \delta_{ij}^{\otimes d+t}. \tag{20}$$

For odd $l_1 + l_2 - l_3 = 2d + 1$:

$$H_{k_1\ldots k_{l_3} i_1\ldots i_{l_1} j_1\ldots j_{l_2}} = D_{j_1 j_2 j_3} \sum_{t=0}^{\min(j_1,j_2)-d-1} (-2)^t \frac{(2j_3-2t-1)!!}{(2j_3-1)!!} \{\epsilon_{kij} \delta_{ik}^{\otimes j_1-(d+t)-1} \delta_{jk}^{\otimes j_2-(d+t)-1} \delta_{kk}^{\otimes t}\} \delta_{ij}^{\otimes k+t}. \tag{21}$$

In computer implementation, one can precompute $\mathbf{H}_{l_1+l_2+l_3}$ (both symbolically and numerically) for different l_1, l_2, and l_3 and store them. Then $\mathbf{Z}_{l_3}$ can be efficiently evaluated by performing contractions between $\mathbf{H}_{l_1+l_2+l_3}$, $\mathbf{X}_{l_1}$, and $\mathbf{Y}_{l_2}$.

Here, the projection tensor $\mathbf{H}_{l_1+l_2+l_3}$ plays the same role as the Clebsch–Gordan coefficients used in spherical tensor products.

Below we give some concrete examples of $\mathbf{H}$ up to $l_1, l_2, l_3 \leq 2$.

For $l_1 = l_2 = l_3 = 2$, we have $d = 1$, and Eq. (20) evaluates to

$$\begin{aligned}
H_{k_1 k_2 i_1 i_2 j_1 j_2} &= C_{222} \sum_{t=0}^{1} (-2)^t \frac{(3-2t)!!}{3!!} \{\delta_{ik}^{\otimes 1-t} \delta_{jk}^{\otimes 1-t} \delta_{kk}^{\otimes t}\} \delta_{ij}^{\otimes 1+t} \\
&= C_{222} \left(\frac{3!!}{3!!} \{\delta_{ik}\delta_{jk}\} \delta_{ij} - \frac{1!!}{3!!} \{\delta_{kk}\} \delta_{ij}^{\otimes 2} \right) \\
&= C_{222} \left(\{\delta_{ik}\delta_{jk}\} \delta_{ij} - \frac{1}{3} \delta_{kk} \delta_{ij}^{\otimes 2} \right) \\
&= C_{222} \left(\delta_{i_1 k_1} \delta_{j_1 k_2} \delta_{i_2 j_2} + \delta_{i_1 k_2} \delta_{j_1 k_1} \delta_{i_2 j_2} - \frac{1}{3} \delta_{k_1 k_2} \delta_{i_1 j_1} \delta_{i_2 j_2} \right)
\end{aligned} \tag{22}$$

We note that $\{\delta_{ik}\delta_{jk}\}\delta_{ij} = \delta_{i_1k_1}\delta_{j_1k_2}\delta_{i_2j_2} + \delta_{i_1k_2}\delta_{j_1k_1}\delta_{i_2j_2}$ because:

- The symmetrization $\{\}$ should be performed only over **unique** permutations.

- i_1 and i_2 are interchangeable since they are associated with the symmetric natural tensor $\mathbf{X}_2$, and, similarly, j_1 and j_2 are interchangeable since they are associated with the symmetric natural tensor $\mathbf{Y}_2$.

Consequently, we only need to symmetrize over the unique permutations of k_1 and k_2.

Other examples can be similarly evaluated. Below we give the results without including the coefficients $C_{l_1l_2l_3}$ and $D_{l_1l_2l_3}$ for simplicity.

When $l_1 = 0$:

$l_2 = 0$, $l_3 = 0$: 1

$l_2 = 1$, $l_3 = 1$: $\delta_{k_1j_1}$

$l_2 = 2$, $l_3 = 2$: $\delta_{k_1j_1}\delta_{k_2j_2}$

When $l_1 = 1$:

$l_2 = 0$, $l_3 = 1$: $\delta_{k_1i_1}$

$l_2 = 1$, $l_3 = 0$: $\delta_{i_1j_1}$

$l_2 = 1$, $l_3 = 1$: $\epsilon_{k_1i_1j_1}$

$l_2 = 1$, $l_3 = 2$: $\delta_{k_1i_1}\delta_{k_2j_1} + \delta_{k_1j_1}\delta_{k_2i_1} - \frac{2}{3}\delta_{k_1k_2}\delta_{i_1j_1}$

$l_2 = 2$, $l_3 = 1$: $\delta_{k_1j_1}\delta_{i_1j_2}$

$l_2 = 2$, $l_3 = 2$: $\delta_{k_1j_2}\epsilon_{k_2i_1j_1} + \delta_{k_2j_2}\epsilon_{k_1i_1j_1}$

When $l_1 = 2$:

$l_2 = 0$, $l_3 = 2$: $\delta_{k_1i_1}\delta_{k_2i_2}$

$l_2 = 1$, $l_3 = 1$: $\delta_{k_1i_1}\delta_{j_1i_2}$

$l_2 = 1$, $l_3 = 2$: $\delta_{k_1i_2}\epsilon_{k_2j_1i_1} + \delta_{k_2i_2}\epsilon_{k_1j_1i_1}$

$l_2 = 2$, $l_3 = 0$: $\delta_{i_1j_1}\delta_{i_2j_2}$

$l_2 = 2$, $l_3 = 1$: $\epsilon_{k_1i_1j_1}\delta_{i_2j_2}$

$l_2 = 2$, $l_3 = 2$: $\delta_{i_1k_1}\delta_{j_1k_2}\delta_{i_2j_2} + \delta_{i_1k_2}\delta_{j_1k_1}\delta_{i_2j_2} - \frac{1}{3}\delta_{k_1k_2}\delta_{i_1j_1}\delta_{i_2j_2}$

1.3. Decomposition and reconstruction of physical tensors

An approach to obtaining the decomposition spectrum of high-rank tensors was proposed by Coope and coworkers [7, 8]. **We extend their approach and propose a systematic procedure for obtaining the decomposition spectrum of physical tensors of arbitrary rank and symmetry.** We extend their work in two aspects: first, we propose to use QR factorization to find linearly independent and orthogonal natural tensor candidates; and second, we introduce a symmetry-informed elimination procedure to identify natural tensor candidates that are compatible with the symmetry of the physical tensor.

1.3.1. Decomposition spectrum of Cartesian tensors

We first briefly review the method by Coope and coworkers [7, 8] to obtain the decomposition spectrum of generic Cartesian tensors. A rank-n Cartesian tensor $\mathbf{T}_n$ can be decomposed into the direct sum of irreducible components (natural tensors) as

$$\mathbf{T}_n = \sum_{\oplus m,p} \mathbf{X}_m^p, \tag{23}$$

where $\mathbf{X}_m^p$ is a natural tensor of rank m (called the weight), and p (called the seniority) is used to distinguish different natural tensors of the same rank [3]. This means there can be multiple natural tensors of the same rank j in the decomposition spectrum of a rank-n Cartesian tensor. Supplementary Table 2 lists the decomposition spectrum of generic Cartesian tensors up to rank seven.

To obtain the natural tensors, one first constructs the natural projector,

$$\mathbf{E}_{2m} = \sum_{t=0}^{\lfloor m/2 \rfloor} c_t \mathbf{I}_{ik}^{\otimes(m-2t)} \mathbf{I}_{ii}^{\otimes t} \mathbf{I}_{kk}^{\otimes t}, \tag{24}$$

that is,

$$E_{k_1 \dots k_m i_1 \dots i_m} = \sum_{t=0}^{\lfloor m/2 \rfloor} c_t (\delta_{ik})^{m-2t} (\delta_{ii})^t (\delta_{kk})^t \tag{25}$$

$\mathbf{E}_{2m}$ is a rank-$2m$ tensor that maps between the space of natural tensors and the space of ordinary Cartesian tensors. Following the notation in Ref. [7], $(\delta_{rr})^t$ is a shorthand notation for a full symmetrization of the product of t Kronecker deltas, each contracting a pair of

Supplementary Table 2: Decomposition spectrum of generic Cartesian tensors up to rank seven. Number of independent irreducible components in the decomposition spectrum of a rank-n tensor for different weight m. For example, a rank-2 Cartesian tensor can be decomposed into a rank-0, rank-1, and a rank-2 natural tensors; a rank-3 Cartesian tensor can be decomposed into a rank-0, three rank-1, two rank-2, and a rank-3 natural tensors. The table is based on Figure 1 in Ref. [7].

		Rank n							
		0	1	2	3	4	5	6	7
Weight m	0	1		1	1	3	6	15	36
	1		1	1	3	6	15	36	91
	2			1	2	6	15	40	105
	3				1	3	10	29	84
	4					1	4	15	49
	5						1	5	21
	6							1	6
	7								1

indices. For example,

$$(\delta_{ik})^2 = \frac{1}{2}(\delta_{i_1k_1}\delta_{i_2k_2} + \delta_{i_1k_2}\delta_{i_2k_1}), \tag{26}$$

$$(\delta_{ii})^2 = \frac{1}{3}(\delta_{i_1i_2}\delta_{i_3i_4} + \delta_{i_1i_3}\delta_{i_2i_4} + \delta_{i_1i_4}\delta_{i_2i_3}), \tag{27}$$

and when $m = 3$ and $t = 1$, we have:

$$\begin{aligned}\delta_{ik}\delta_{ii}\delta_{kk} =& \frac{1}{9}(\delta_{i_1k_1}\delta_{i_2i_3}\delta_{k_2k_3} + \delta_{i_1k_2}\delta_{i_2i_3}\delta_{k_1k_3} + \delta_{i_1k_3}\delta_{i_2i_3}\delta_{k_1k_2} \\ &+ \delta_{i_2k_1}\delta_{i_1i_3}\delta_{k_2k_3} + \delta_{i_2k_2}\delta_{i_1i_3}\delta_{k_1k_3} + \delta_{i_2k_3}\delta_{i_1i_3}\delta_{k_1k_2} \\ &+ \delta_{i_3k_1}\delta_{i_1i_2}\delta_{k_2k_3} + \delta_{i_3k_2}\delta_{i_1i_2}\delta_{k_1k_3} + \delta_{i_3k_3}\delta_{i_1i_2}\delta_{k_1k_2}).\end{aligned} \tag{28}$$

The coefficient c_t is defined as:

$$c_t = (-1)^t \frac{(m!)^2}{(2m)!}\binom{m}{t}\binom{2m-2t}{m}, \tag{29}$$

where $\binom{a}{b}$ is the binomial coefficient.

Eq. (24) can essentially be used to project out the rank m natural tensor from a rank-m Cartesian tensor. When $t = 0$, it symmetrizes the Cartesian tensor, and when $t > 0$, it removes the traces.

The projector $\mathbf{G}^p_{m+n}$ can be seen as a generalization of $\mathbf{H}_{2n}$ in Eq. (6) to tensors with arbitrary symmetries: Eq. (6) applies only to completely symmetric tensors such as $\mathbf{U}$, whereas $\mathbf{G}^p_{m+n}$ additionally handles the symmetrization of general tensors (via the $t = 0$ contribution in $\mathbf{E}_{2m}$) before removing traces. Since $\mathbf{U}$ is already fully symmetric, only trace removal is needed, making Eq. (6) more concise.

With $\mathbf{E}_{2m}$, we can also construct the projectors that map between a general tensor $\mathbf{T}_n$ and the natural tensor $\mathbf{X}^p_m$ of weight m. First, the projector tensor $\mathbf{G}^p_{m+n}$ that embeds a natural tensor $\mathbf{X}^p_m$ into a Cartesian tensor space of rank n is constructed as follows.

For even $n - m$,

$$\mathbf{G}^p_{m+n} = \mathbf{E}_{2m} \otimes \mathbf{I}^{\otimes(n-m)/2} \qquad G^p_{k_1 \ldots k_m i_1 \ldots i_n} = E_{k_1 \ldots k_m i_1 \ldots i_m} (\delta_{ii})^{(n-m)/2}, \tag{30}$$

For odd $n - m$,

$$\mathbf{G}^p_{m+n} = \mathbf{E}_{2m} \odot \boldsymbol{\epsilon} \otimes \mathbf{I}^{\otimes(n-m-1)/2} \quad G^p_{k_1 \ldots k_m i_1 \ldots i_n} = E_{k_1 \ldots k_m i_1 \ldots i_m} \epsilon_{i_m i_{m+1} i_{m+2}} (\delta_{ii})^{(n-m-1)/2}. \tag{31}$$

$\mathbf{G}^p_{m+n}$ has the following property:

$$\mathbf{G}^p_{m+n} \odot^n \mathbf{G}^q_{m+n} = g_{pq} \mathbf{E}_{2m}, \tag{32}$$

where g_{pq} is a scalar. All the values of g_{pq} of different seniority p and q form a symmetric matrix $\mathbf{g}$, and its inverse is

$$\mathbf{h} = \mathbf{g}^{-1}. \tag{33}$$

With these, we can construct the natural tensor extractor $\mathbf{H}^p_{(m|n)}$, which extracts the natural tensor $\mathbf{X}^p_m$ from a Cartesian tensor $\mathbf{T}_n$. It is defined as

$$\mathbf{H}^p_{m+n} = \sum_q h_{pq} \mathbf{G}^q_{m+n}. \tag{34}$$

The above discussion concludes the construction of the projector tensors $\mathbf{G}^p_{m+n}$ and $\mathbf{H}^p_{m+n}$ to decompose and reconstruct natural tensors of weight m from a rank-n Cartesian tensor. Let us reiterate the key results:

Given a rank-n Cartesian tensor $\mathbf{T}_n$, we can extract the natural tensor $\mathbf{X}_m^p$ of rank m as

$$\mathbf{X}_m^p = \mathbf{H}_{m+n}^p \odot^n \mathbf{T}_n; \tag{35}$$

Given a natural tensor $\mathbf{X}_m$ of weight m, we can embed it into a tensor space of rank-n by

$$\mathbf{S}_n^p = \mathbf{G}_{m+n}^p \odot^m \mathbf{X}_m; \tag{36}$$

Consequently, a rank-n Cartesian tensor $\mathbf{T}_n$ can be reconstructed from its set of natural representations $\mathbf{X}_m^p$ as

$$\mathbf{T}_n = \sum_{m,p} \mathbf{G}_{m+n}^p \odot^m \mathbf{X}_m^p. \tag{37}$$

1.3.2. Selection of independent natural tensors

The above procedure provides a way to construct the projector tensors $\mathbf{G}_{m+n}^p$ and $\mathbf{H}_{m+n}^p$, which can then be used to decompose a Cartesian tensor into natural tensors (Eq. (35)), and, conversely, reconstruct a Cartesian tensor from its natural representations (Eq. (37)). The number of candidate projector tensors constructed this way, however, is often larger than the actual number of independent natural tensors. For example, for a rank-3 Cartesian tensor, the above procedure gives three candidates of rank-2 projectors, but only two of them are independent as seen in Supplementary Table 2. Explicitly, when $n = 3$ and $m = 2$, from Eq. (31), we have $\mathbf{G}_5 = \mathbf{E}_4 \odot \boldsymbol{\epsilon}$. There are three candidate projectors:

$$\begin{aligned} G^1_{k_1k_2i_1i_2i_3} &= E_{k_1k_2i_1i_\alpha}\epsilon_{i_\alpha i_2 i_3} \\ G^2_{k_1k_2i_1i_2i_3} &= E_{k_1k_2i_1i_\alpha}\epsilon_{i_2 i_\alpha i_3}, \\ G^3_{k_1k_2i_1i_2i_3} &= E_{k_1k_2i_1i_\alpha}\epsilon_{i_2 i_3 i_\alpha} \end{aligned} \tag{38}$$

depending on which index i_α of the Levi-Civita symbol $\boldsymbol{\epsilon}$ is contracted with the natural projector $\mathbf{E}_4$. But they are linearly dependent, and only two of them are independent. We can select any two of them as the independent projectors, and the third one can be expressed as a linear combination of the two independent ones.

The identification of linearly independent $\mathbf{G}_{m+n}^p$ (p is an index for different candidates) can be done as follows:

1. Create an arbitrary natural tensor $\mathbf{X}_m$ of weight m;

2. Embed $\mathbf{X}_m$ into the space of rank-n Cartesian tensors via Eq. (36) for all candidates.

3. Find a set of linearly independent $\mathbf{S}_n^p$, and the corresponding $\mathbf{G}_{m+n}^p$ are the independent projectors.

Step 3 above can be conducted using the QR factorization [9]. Specifically, we represent the tensor $\mathbf{S}_n^p$ as a vector $\mathbf{s}^p$ of length 3^n and construct a 3^n by p matrix $\mathbf{A}$ by putting each $\mathbf{s}^p$ as a column. Then, a QR factorization of $\mathbf{A}$ is performed to identify its linearly independent columns, and the corresponding $\mathbf{G}_{m+n}^p$ are the independent projectors.

1.3.3. Decomposition of symmetric Cartesian tensors

Supplementary Table 3: Decomposition spectrum of the physical tensors with inherent symmetry. Indices in a pair of parentheses are symmetric, and those in a pair of double parentheses are symmetric with respect to exchange of the two pairs.

Rank n	Symmetry	Example	# independent components	Spectrum l: 0	1	2	3	4
0		Energy	1	1				
1	i	Dipole moment / Forces	3		1			
2	ij	Nuclear shielding	9	1	1	1		
	(ij)	Polarizability	6	1		1		
4	ijkl	Generic rank-4 tensor	81	3	6	6	3	1
	((ij)(kl))	Elasticity	21	2		2		1

So far, we have discussed the decomposition of generic Cartesian tensors. Many physical tensors, however, have inherent symmetries. For example, the polarizability is a symmetric rank-2 tensor, and its decomposition spectrum contains only a rank-0 and a rank-2 natural tensor (Supplementary Table 3), without the rank-1 part for a generic rank-2 tensor (Supplementary Table 2).

For tensors with inherent symmetries, the number of independent natural tensors in their decomposition spectrum is fewer than that of generic Cartesian

tensors. Thus, the number of projectors $\mathbf{G}^p_{m+n}$ and $\mathbf{H}^p_{(m|n)}$ should be reduced accordingly. Here, we propose a systematic procedure to identify the independent projectors for physical tensors with inherent symmetries.

1. **Obtain all independent $\mathbf{G}^p_{m+n}$ as discussed in "Selection of independent natural tensors", where p is an index for different candidates ($p = 1, 2, \ldots, N$).**

2. **Identify a subset of N_g unique linearly independent mapping tensors from all N candidates $\mathbf{G}^1, \ldots, \mathbf{G}^N$.** Create a rank-$n$ tensor $\mathbf{T}_n$ with the target symmetry, and then compute $\mathbf{F}^p_n = \mathbf{G}^p_{m+n} \odot^n \mathbf{T}_n$ for all independent $\mathbf{G}^p_{m+n}$. Because there is inherent symmetry in $\mathbf{T}_n$, some $\mathbf{F}^p_n$ will be identical to others. Next, group the $\mathbf{G}^p_{m+n}$ according to $\mathbf{F}^p_n$: the $\mathbf{G}^p_{m+n}$ that give the same $\mathbf{F}^p_n$ are equivalent and are put in the same group. Let N_g be the number of groups; then $N_d = N - N_g$ is the number of dependent $\mathbf{G}^p_{m+n}$.

3. **Construct the decomposition projector H to extract natural tensors from a physical tensor with inherent symmetry.** Compute $\mathbf{H}^p_{m+n} = \sum_q h_{pq}\mathbf{G}^q_{m+n}$ (i.e., Eq. (34)) for $p = 1, 2, \ldots, N$, where $q = 1, 2, \ldots, N$. In total, there are N of $\mathbf{H}^p_{m+n}$, but only N_g of them are independent, since they are linear combinations of $\mathbf{G}^p_{m+n}$ and there are only N_g unique $\mathbf{G}^p_{m+n}$ as identified in step 2. Without loss of generality, let the first N_g of $\mathbf{H}^p_{(j|n)}$ be the independent ones, and the remaining N_d of them be the dependent ones (If not, we can reindex them). Then, we can express the dependent $\mathbf{H}^q_{m+n}$ as linear combinations of the independent ones: $\mathbf{H}^q_{m+n} = \sum_{p=1}^{N_g} \beta_{qp}\mathbf{H}^p_{m+n}$, where $q = N_g+1, N_g+2, \ldots, N$. The coefficients β_{qp} can be solved by combining this equation and Eq. (34).

4. **Construct the reconstruction tensor Q to embed natural tensors into the space of physical tensor with inherent symmetry.** We create $\mathbf{Q}^p_{m+n} = \mathbf{G}^p_{m+n} + \sum_{q=N_g+1}^{N} \beta_{qp}\mathbf{G}^q_{m+n}$, where $p = 1, 2, \ldots, N_g$.

With $\mathbf{H}^p_{m+n}$ obtained in step 3 and $\mathbf{Q}^p_{m+n}$ obtained in step 4 (where $p = 1, 2, \ldots, N_g$), one can use Eqs. (35), (36), and (37) to extract the natural tensors from a physical tensor with inherent symmetry, and embed the natural tensors into the space of physical tensor with inherent symmetry. Consequently, this allows for the mapping between natural tensors and physical tensors of arbitrary rank and with any inherent symmetry.

1.3.4. Example for polarizability

Using the polarizability tensor as an example, we illustrate the above procedure to identify the independent projectors for a symmetric rank-2 tensor. As shown in Supplementary Table 2, a generic rank-2 Cartesian tensor can be decomposed into a rank-0, a rank-1, and a rank-2 natural tensors, but a symmetric rank-2 tensor does not have the rank-1 part in its decomposition spectrum, and its decomposition spectrum only consists of a rank-0 and a rank-2 natural tensors (Supplementary Table 3).

Let's get the projector to extract the rank-0 natural tensor.

Step 1, using Eq. (30), when $n = 2$ and $m = 0$, we have

$$G^1_{i_1 i_2} = E_0 \delta_{i_1 i_2} = 1 \cdot \delta_{i_1 i_2} = \delta_{i_1 i_2}. \tag{39}$$

Step 2, given that there is a single candidate G, it is independent by default, and thus $N_g = 1$ and $N_d = 0$.

Step 3, $\mathbf{G}^1 \odot^2 \mathbf{G}^1 = \delta_{i_1 i_2}\delta_{i_1 i_2} = 3$. Then according to Eq. (32) and Eq. (33), we have $g_{11} = 3$ and $h_{11} = g_{11}^{-1} = 1/3$. Therefore, according to Eq. (34), we have $H^1_{i_1 i_2} = h_{11} G^1_{i_1 i_2} = \frac{1}{3}\delta_{i_1 i_2}$.

Step 4, given that there is a single candidate G, we have $Q^1_{i_1 i_2} = G^1_{i_1 i_2} = \delta_{i_1 i_2}$.

Next, let's get the projector to extract the rank-2 natural tensor.

Step 1, using Eq. (30), when $n = 2$ and $m = 2$, we have

$$\begin{aligned} G_{k_1 k_2 i_1 i_2} = E_{k_1 k_2 i_1 i_2} &= \sum_{t=0}^{1} c_t (\delta_{ik})^{2-2t} (\delta_{ii})^t (\delta_{kk})^t \\ &= c_0 (\delta_{ik})^2 + c_1 (\delta_{ii})(\delta_{kk}) = \frac{1}{2}(\delta_{i_1 k_1}\delta_{i_2 k_2} + \delta_{i_1 k_2}\delta_{i_2 k_1}) - \frac{1}{3}\delta_{i_1 i_2}\delta_{k_1 k_2} \end{aligned} \tag{40}$$

where in the second equation, we have used Eq. (25), and $c_0 = 1$ and $c_1 = -\frac{1}{3}$ are computed using the formula of c_t in Eq. (29).

Step 2, given that there is a single candidate G, it is independent by default, and thus $N_g = 1$ and $N_d = 0$.

Step 3, $\mathbf{G}^1 \odot^2 \mathbf{G}^1 = G_{k_1 k_2 i_1 i_2} G_{k_3 k_4 i_1 i_2} = [\frac{1}{2}(\delta_{i_1 k_1}\delta_{i_2 k_2} + \delta_{i_1 k_2}\delta_{i_2 k_1}) - \frac{1}{3}\delta_{i_1 i_2}\delta_{k_1 k_2}] \cdot [\frac{1}{2}(\delta_{i_1 k_3}\delta_{i_2 k_4} + \delta_{i_1 k_4}\delta_{i_2 k_3}) - \frac{1}{3}\delta_{i_1 i_2}\delta_{k_3 k_4}] = [\frac{1}{2}(\delta_{k_1 k_3}\delta_{k_2 k_4} + \delta_{k_1 k_4}\delta_{k_2 k_3}) - \frac{1}{3}\delta_{k_1 k_2}\delta_{k_3 k_4}]$. Then according to Eq. (32) and Eq. (33), we have $g_{11} = 1$ and $h_{11} = g_{11}^{-1} = 1$. Then according to Eq. (34), we have $H^1_{k_1 k_2 i_1 i_2} = h_{11} G^1_{k_1 k_2 i_1 i_2} = G^1_{k_1 k_2 i_1 i_2}$.

Step 4, given that there is a single candidate G, we have $Q^1_{k_1 k_2 i_1 i_2} = G^1_{k_1 k_2 i_1 i_2}$.

Supplementary Note 2. Comparison with other Cartesian methods

There are several existing equivariant Cartesian methods for atomistic ML. According to the way the internal equivariant features are constructed, they can be categorized into two groups: those that use reducible Cartesian tensors as features (including MTP [10], FieldSchNet [11], CAMP [12], and HotPP [13]) and those that use irreducible Cartesian tensors as features (including REANN [14], CACE [15], TensorNet [16], ICTP [17], and TACE [18]).

Methods using reducible Cartesian tensors, such as CAMP and HotPP, typically operate on full Cartesian products that satisfy $E(3)$ equivariance but cannot respect the intrinsic symmetries inherent in many physical tensors. As a concrete example, a rank-4 elastic constant tensor C_{ijkl} contains $3^4 = 81$ components, yet it possesses only 21 independent components once minor and major symmetries ($C_{ijkl} = C_{jikl} = C_{klij}$) are considered. While models like HotPP must parameterize and model all 81 components, CarNet automatically utilizes irreducible Cartesian tensors to work directly within the symmetry-allowed manifold. By focusing only on the 21 independent components, CarNet eliminates mathematical redundancy and achieves a more compact representation without sacrificing geometric expressiveness.

Existing methods using irreducible Cartesian tensors address symmetry issues but lack the generality to systematically handle tensors of arbitrary ranks and symmetries. REANN and CACE focus exclusively on scalar targets. TensorNet advances this by modeling targets up to rank-2, yet its internal features are restricted to rank-0, 1, and 2, with no systematic method provided for higher-rank extension. While ICTP and the recent TACE are more similar to CarNet in their use of irreducible Cartesian internal features, they face distinct limitations. Compared to CarNet, ICTP relies on inefficient loops to construct natural tensors and perform tensor products (i.e., Eqs. (2), (11), and (12)), whereas CarNet utilizes an efficient projector-based approach. Moreover, ICTP and TACE lack a systematic framework to identify independent natural tensors or construct projectors for high-rank targets with complex inherent symmetries. In principle, extending these methods to arbitrary ranks is possible, but no formal theory or practical implementation for handling high-rank tensors with complex inherent symmetries currently exists. Consequently, these models have only

been demonstrated on targets up to the straightforward rank-2, where symmetry constraints are relatively straightforward to manage. In contrast, this work establishes a general Cartesian model based on three essential components: (1) constructing natural tensors from unit vectors, (2) performing tensor products between natural tensors, and (3) decomposing and reconstructing physical tensors of arbitrary rank with any inherent symmetry. This third component fills the gap existing in ICTP and TACE.

Supplementary Note 3. Nuclear chemical shifts

The nuclear chemical shifts can be obtained from the nuclear shielding tensor as

$$\delta = \sigma_{\text{ref}}^{Z} - \frac{1}{3}\text{Tr}[\boldsymbol{\sigma}], \tag{41}$$

where Tr denotes the trace of a rank-2 tensor, and σ_{ref}^{Z} is the reference shifts for a tetramethylsilane molecule. The reference shifts computed with the PBE0/def2-TZVP functional are $\sigma^{\text{H}} = 31.77$ ppm and $\sigma^{\text{C}} = 188.53$ ppm [19].

The nuclear shielding tensor $\boldsymbol{\sigma}$ in the original ethanol dataset [19] is originally given in atomic units (a.u.). To convert it to ppm, a conversion factor c is multiplied:

$$c = \alpha^2 \times 10^6 = 53.25135, \tag{42}$$

where

$$\alpha = \frac{e^2}{4\pi\epsilon_0\hbar c} \approx 0.007297 \tag{43}$$

is the fine-structure constant.

Supplementary Table 4: Additional results on learning nuclear shielding of ethanol. Mean absolute errors (MAEs) of the nuclear shielding tensor for all elements $\boldsymbol{\sigma}_{\text{all}}$, hydrogen $\boldsymbol{\sigma}_{\text{H}}$, carbon $\boldsymbol{\sigma}_{\text{C}}$, and oxygen $\boldsymbol{\sigma}_{\text{O}}$, as well as the corresponding chemical shift δ_{all}, δ_{H}, δ_{C}, and δ_{O} are reported. Chemical shift is computed as one third of the trace of the shielding tensor, with a constant offset.

	$\boldsymbol{\sigma}_{\text{all}}$	$\boldsymbol{\sigma}_{\text{H}}$	$\boldsymbol{\sigma}_{\text{C}}$	$\boldsymbol{\sigma}_{\text{O}}$	δ_{all}	δ_{H}	δ_{C}	δ_{O}
FieldSchNet					0.169	0.123	0.194	0.401
TensorNet					0.139			
CarNet	0.047	0.021	0.066	0.166	0.031	0.013	0.048	0.105

Supplementary Note 4. Tensor product modes

For a tensor product $\mathbf{Z}_{l_3} = \mathbf{X}_{l_1} \hat{\otimes} \mathbf{Y}_{l_2}$, different combinations of l_1 and l_2 can lead to the same output tensor of rank l_3. The 3-tuple (l_1, l_2, l_3) is called a path. According to the product rule of natural tensors for a given l_1 and l_2, all l_3 satisfying

$$|l_1 - l_2| \leq l_3 \leq l_1 + l_2 \tag{44}$$

are allowed values. In building the models, we restrict the maximum rank of the used tensors to L, meaning that

$$0 \leq l_1, l_2, l_3 \leq L. \tag{45}$$

'Full' mode. All paths (l_1, l_2, l_3) satisfying Eqs. (44) and (45) are allowed.

'Lite' mode. In addition to Eqs. (44) and (45), the paths are further restricted to those with

$$l_3 = |l_1 - l_2|. \tag{46}$$

This is inspired by the tensor contraction rule in the CAMP model [12], where all indices in $\mathbf{X}_{l_1}$ are contracted away by the indices in $\mathbf{Y}_{l_2}$. It is required that $l_1 \leq l_2$, and the output is $\mathbf{Z}_{l_2 - l_1} = \mathbf{X}_{l_1} \odot^{l_1} \mathbf{Y}_{l_2}$, where $\odot^{l_1}$ denotes an l_1-th order contraction. As can be seen, this operation is not symmetric, since the rank l_1 has to be smaller than the rank l_2; in other words, the order of $\mathbf{X}_{l_1}$ and $\mathbf{Y}_{l_2}$ matters. Here, we improve this by allowing for a symmetric operation according to Eq. (46), which allows for both $l_1 \leq l_2$ and $l_1 \geq l_2$.

'Level' mode. Eqs. (44) and (45) are used for scalar output $l_3 = 0$. But for $l_3 > 0$, in addition to the two equations, the paths are further restricted to those with

$$l_1 + l_2 \leq l_l, \tag{47}$$

where l_l is an integer in the range of $[L, 2L]$. This mode provides a way to control the complexity of the model by tuning the level l_l. When l_l is chosen to have a value of $2L$, this is equivalent to the 'full' mode. For other values, it further restricts the tensor products to be between tensors of smaller ranks. **We use $l_l = L$ for all results reported in this work.**

Supplementary Tables 5, 6, and 7 explicitly list the paths for the 'full', 'lite', and 'level' models for $L = 2$, $L = 3$, and $L = 4$, respectively. It should be clear that:

1. The full mode has many more paths for larger l_3 than for smaller l_3, particularly when L is large.

2. The lite mode has roughly the same number of paths for different l_3. This is also the case for the level mode.

3. The total number of paths for the lite mode and the level model are about the same.

Supplementary Table 5: Tensor product paths for $L = 2$

**Mode**	l_3	**# Paths**	**Paths** (l_1, l_2, l_3)
full	0	3	(0, 0, 0), (1, 1, 0), (2, 2, 0)
	1	6	(0, 1, 1), (1, 0, 1), (1, 1, 1), (1, 2, 1), (2, 1, 1), (2, 2, 1)
	2	6	(0, 2, 2), (1, 1, 2), (1, 2, 2), (2, 0, 2), (2, 1, 2), (2, 2, 2)
lite	0	3	(0, 0, 0), (1, 1, 0), (2, 2, 0)
	1	4	(0, 1, 1), (1, 0, 1), (1, 2, 1), (2, 1, 1)
	2	2	(0, 2, 2), (2, 0, 2)
level	0	3	(0, 0, 0), (1, 1, 0), (2, 2, 0)
	1	3	(0, 1, 1), (1, 0, 1), (1, 1, 1)
	2	3	(0, 2, 2), (1, 1, 2), (2, 0, 2)

Supplementary Table 6: Tensor product paths for $L = 3$

**Mode**	l_3	**# Paths**	**Paths** (l_1, l_2, l_3)
full	0	4	(0, 0, 0), (1, 1, 0), (2, 2, 0), (3, 3, 0)
	1	9	(0, 1, 1), (1, 0, 1), (1, 1, 1), (1, 2, 1), (2, 1, 1), (2, 2, 1), (2, 3, 1), (3, 2, 1) (3, 3, 1)
	2	11	(0, 2, 2), (1, 1, 2), (1, 2, 2), (1, 3, 2), (2, 0, 2), (2, 1, 2), (2, 2, 2), (2, 3, 2) (3, 1, 2), (3, 2, 2), (3, 3, 2)
	3	10	(0, 3, 3), (1, 2, 3), (1, 3, 3), (2, 1, 3), (2, 2, 3), (2, 3, 3), (3, 0, 3), (3, 1, 3) (3, 2, 3), (3, 3, 3)
lite	0	4	(0, 0, 0), (1, 1, 0), (2, 2, 0), (3, 3, 0)
	1	6	(0, 1, 1), (1, 0, 1), (1, 2, 1), (2, 1, 1), (2, 3, 1), (3, 2, 1)
	2	4	(0, 2, 2), (1, 3, 2), (2, 0, 2), (3, 1, 2)
	3	2	(0, 3, 3), (3, 0, 3)
level	0	4	(0, 0, 0), (1, 1, 0), (2, 2, 0), (3, 3, 0)
	1	5	(0, 1, 1), (1, 0, 1), (1, 1, 1), (1, 2, 1), (2, 1, 1)
	2	5	(0, 2, 2), (1, 1, 2), (1, 2, 2), (2, 0, 2), (2, 1, 2)
	3	4	(0, 3, 3), (1, 2, 3), (2, 1, 3), (3, 0, 3)

Supplementary Table 7: Tensor product paths for $L = 4$

Mode	l_3	# Paths	Paths (l_1, l_2, l_3)
full	0	5	(0, 0, 0), (1, 1, 0), (2, 2, 0), (3, 3, 0), (4, 4, 0)
	1	12	(0, 1, 1), (1, 0, 1), (1, 1, 1), (1, 2, 1), (2, 1, 1), (2, 2, 1), (2, 3, 1), (3, 2, 1) (3, 3, 1), (3, 4, 1), (4, 3, 1), (4, 4, 1)
	2	16	(0, 2, 2), (1, 1, 2), (1, 2, 2), (1, 3, 2), (2, 0, 2), (2, 1, 2), (2, 2, 2), (2, 3, 2) (2, 4, 2), (3, 1, 2), (3, 2, 2), (3, 3, 2), (3, 4, 2), (4, 2, 2), (4, 3, 2), (4, 4, 2)
	3	17	(0, 3, 3), (1, 2, 3), (1, 3, 3), (1, 4, 3), (2, 1, 3), (2, 2, 3), (2, 3, 3), (2, 4, 3) (3, 0, 3), (3, 1, 3), (3, 2, 3), (3, 3, 3), (3, 4, 3), (4, 1, 3), (4, 2, 3), (4, 3, 3) (4, 4, 3)
	4	15	(0, 4, 4), (1, 3, 4), (1, 4, 4), (2, 2, 4), (2, 3, 4), (2, 4, 4), (3, 1, 4), (3, 2, 4) (3, 3, 4), (3, 4, 4), (4, 0, 4), (4, 1, 4), (4, 2, 4), (4, 3, 4), (4, 4, 4)
lite	0	5	(0, 0, 0), (1, 1, 0), (2, 2, 0), (3, 3, 0), (4, 4, 0)
	1	8	(0, 1, 1), (1, 0, 1), (1, 2, 1), (2, 1, 1), (2, 3, 1), (3, 2, 1), (3, 4, 1), (4, 3, 1)
	2	6	(0, 2, 2), (1, 3, 2), (2, 0, 2), (2, 4, 2), (3, 1, 2), (4, 2, 2)
	3	4	(0, 3, 3), (1, 4, 3), (3, 0, 3), (4, 1, 3)
	4	2	(0, 4, 4), (4, 0, 4)
level	0	5	(0, 0, 0), (1, 1, 0), (2, 2, 0), (3, 3, 0), (4, 4, 0)
	1	6	(0, 1, 1), (1, 0, 1), (1, 1, 1), (1, 2, 1), (2, 1, 1), (2, 2, 1)
	2	8	(0, 2, 2), (1, 1, 2), (1, 2, 2), (1, 3, 2), (2, 0, 2), (2, 1, 2), (2, 2, 2), (3, 1, 2)
	3	7	(0, 3, 3), (1, 2, 3), (1, 3, 3), (2, 1, 3), (2, 2, 3), (3, 0, 3), (3, 1, 3)
	4	5	(0, 4, 4), (1, 3, 4), (2, 2, 4), (3, 1, 4), (4, 0, 4)

Supplementary Note 5. Algorithm to compute hyper moment

Algorithm 1 Iterative Calculation of Hyper Moment Tensors

Require: Atomic moment M_{ul}; Maximum correlation degree $v_{\max}$

$\mathbf{H}_{ul}^{1} \leftarrow \mathbf{M}_{ul}$

for $v \leftarrow 2$ to $v_{\max}$ **do**

    $\mathbf{H}_{ul_3,p}^{v} \leftarrow \mathbf{H}_{ul_1}^{v-1} \hat{\otimes} \mathbf{M}_{ul_2}$

    $\mathbf{H}_{ul}^{v} \leftarrow \sum_{p} W_p \mathbf{H}_{ul,p}^{v}$

end for

$\mathbf{H}_{ul} \leftarrow \sum_{v=1}^{v_{\max}} W_l^v \mathbf{H}_{ul}^{v}$

SUPPLEMENTARY REFERENCES

[1] M. Wen, M. K. Horton, J. M. Munro, P. Huck, and K. A. Persson, An equivariant graph neural network for the elasticity tensors of all seven crystal systems, Digital Discovery **3**, 869 (2024).

[2] A. D. Kaplan, R. Liu, J. Qi, T. W. Ko, B. Deng, J. Riebesell, G. Ceder, K. A. Persson, and S. P. Ong, A foundational potential energy surface dataset for materials, arXiv preprint arXiv:2503.04070 10.48550/arXiv.2503.04070 (2025).

[3] J. Jerphagnon, D. Chemla, and R. Bonneville, The description of the physical properties of condensed matter using irreducible tensors, Advances in Physics **27**, 609 (1978).

[4] D. R. Lehman and W. C. Parke, Angular reduction in multiparticle matrix elements, Journal of Mathematical Physics **30**, 2797 (1989).

[5] J. A. R. Coope, Irreducible cartesian tensors. iii. clebsch-gordan reduction, Journal of Mathematical Physics **11**, 1591 (1970).

[6] A. Edmonds, *Angular Momentum in Quantum Mechanics*, Investigations in Physics Series (Princeton University Press, 1996).

[7] J. A. R. Coope, R. F. Snider, and F. R. McCourt, Irreducible cartesian tensors, Journal of Chemical Physics **43**, 2269 (1965).

[8] J. A. R. Coope and R. F. Snider, Irreducible cartesian tensors. ii. general formulation, Journal of Mathematical Physics **11**, 1003 (1970).

[9] G. H. Golub and C. F. Van Loan, *Matrix Computations* (Johns Hopkins University Press, Baltimore, MD, USA, 2013).

[10] A. V. Shapeev, Moment tensor potentials: A class of systematically improvable interatomic potentials, Multiscale Modeling & Simulation 10.1137/15M1054183 (2016).

[11] K. Schütt, O. Unke, and M. Gastegger, Equivariant message passing for the prediction of tensorial properties and molecular spectra, in *International Conference on Machine Learning*, Vol. 139 (PMLR, 2021) pp. 9377–9388.

[12] M. Wen, W.-F. Huang, J. Dai, and S. Adhikari, Cartesian atomic moment machine learning interatomic potentials, npj Computational Materials **11**, 128 (2025).

[13] J. Wang, Y. Wang, H. Zhang, Z. Yang, Z. Liang, J. Shi, H.-T. Wang, D. Xing, and J. Sun, E(n)-equivariant cartesian tensor message passing interatomic potential, Nature Communications

15, 10.1038/s41467-024-51886-6 (2024).

[14] Y. Zhang, J. Xia, and B. Jiang, Physically motivated recursively embedded atom neural networks: incorporating local completeness and nonlocality, Physical Review Letters **127**, 156002 (2021).

[15] B. Cheng, Cartesian atomic cluster expansion for machine learning interatomic potentials, npj Computational Materials **10**, 1 (2024).

[16] G. Simeon and G. De Fabritiis, Tensornet: Cartesian tensor representations for efficient learning of molecular potentials, in *Advances in Neural Information Processing Systems*, Vol. 36, edited by A. Oh, T. Naumann, A. Globerson, K. Saenko, M. Hardt, and S. Levine (2023) pp. 37334–37353.

[17] V. Zaverkin, F. Alesiani, T. Maruyama, F. Errica, H. Christiansen, M. Takamoto, N. Weber, and M. Niepert, Higher-rank irreducible cartesian tensors for equivariant message passing, Advances in Neural Information Processing Systems **37**, 124025 (2024).

[18] Z. Xu, W. Xie, and P. Hu, Spectral/spatial tensor atomic cluster expansion with universal embeddings in cartesian space, arXiv preprint arXiv:2509.14961 10.48550/arXiv.2509.14961 (2025).

[19] M. Gastegger, K. T. Schütt, and K.-R. Müller, Machine learning of solvent effects on molecular spectra and reactions, Chemical Science **12**, 11473 (2021).
