# Supplementary material for: Atomistic machine learning with irreducible Cartesian natural tensors
Source: Nat Commun. 2026 Aug 27;17:10282. doi: 10.1038/s41467-026-77263-z (PMC13619602; doi:10.1038/s41467-026-77263-z)
Supplement: Supplementary file 3 [file 41467_2026_77263_MOESM3_ESM.pdf]

nature portfolio

Peer Review File

Atomistic Machine Learning with Irreducible Cartesian Natural Tensors

Corresponding Author: Professor Mingjian Wen

This manuscript has been previously reviewed at another journal. This document only contains information relating to versions considered at Nature Communications.

This file contains all reviewer reports in order by version, followed by all author rebuttals in order by version.

Version 0:

Reviewer comments:

Reviewer #1

(Remarks to the Author)
I reviewed the earlier versions of this manuscript. The authors focused on rank-4 tensors, which I think are less commonly used in diverse scenarios. They have now provided additional results that the proposed model,CarNet, not only advances the learning of high-rank tensors, but also provides improved performance for low-rank tensors such as dipole moment, polarizability, and chemical shielding tensor that are widely used in many spectroscopic applications. I have no more concerns and would love to recommend the publication of this work.

(Remarks on code availability)

Reviewer #2

(Remarks to the Author)
I appreciate the authors for the revised manuscript. My concerns have been addressed, and I recommend the acceptance of the manuscript to Nature Communications.

(Remarks on code availability)
The code is clean and well-documented.

Open Access This Peer Review File is licensed under a Creative Commons Attribution 4.0 International License, which permits use, sharing, adaptation, distribution and reproduction in any medium or format, as long as you give appropriate credit to the original author(s) and the source, provide a link to the Creative Commons license, and indicate if changes were made.
In cases where reviewers are anonymous, credit should be given to 'Anonymous Referee' and the source.
The images or other third party material in this Peer Review File are included in the article's Creative Commons license, unless indicated otherwise in a credit line to the material. If material is not included in the article's Creative Commons license and your intended use is not permitted by statutory regulation or exceeds the permitted use, you will need to obtain permission directly from the copyright holder.
To view a copy of this license, visit https://creativecommons.org/licenses/by/4.0/

Reviewer 1

1. **Comment**: I reviewed the earlier versions of this manuscript. The authors focused on rank-4 tensors, which I think are less commonly used in diverse scenarios. They have now provided additional results that the proposed model, CarNet, not only advances the learning of high-rank tensors, but also provides improved performance for low-rank tensors such as dipole moment, polarizability, and chemical shielding tensor that are widely used in many spectroscopic applications. I have no more concerns and would love to recommend the publication of this work.

   Response: Thank you for your time and recommendation for publication.

Reviewer 2

1. **Comment**: I appreciate the authors for the revised manuscript. My concerns have been addressed, and I recommend the acceptance of the manuscript to Nature Communications.

   Response: Thank you for your time and recommendation for acceptance.

2. **Comment on code availability**: The code is clean and well-documented.

   Response: Thank you for your assessment of the code.
